# Mapping lock-ins and enabling environments for agri-food sustainability transitions in Europe

**DOI:** 10.1007/s11625-024-01480-y

**Published:** 2024-04-08

**Authors:** Tim G. Williams, Matthias Bürgi, Niels Debonne, Vasco Diogo, Julian Helfenstein, Christian Levers, Franziska Mohr, Anne Elise Stratton, Peter H. Verburg

**Affiliations:** 1 Environmental Geography Group, IVM Institute for Environmental Studies, VU University Amsterdam, de Boelelaan 1087, 1081 HV Amsterdam, The Netherlands; 2 Land Change Science Research Unit, Swiss Federal Research Institute WSL, Zurich, Switzerland; 3 Agroecology and Environment, Agroscope, Zurich, Switzerland; 4 Soil Geography and Landscape, Wageningen University, Wageningen, The Netherlands; 5 Thünen Institute of Biodiversity, Johann Heinrich Von Thünen Institute-Federal Research Institute for Rural Areas, Forestry, and Fisheries, Braunschweig, Germany; 6 Sustainable Use of Natural Resources Department, Institute of Social Sciences in Agriculture, University of Hohenheim, Stuttgart, Germany

**Keywords:** Agriculture, Farming, Governance, Spatial analysis, Socioeconomic indicators, Transformation

## Abstract

European agri-food systems must overcome structural lock-ins to achieve more sustainable modes of production and consumption. Yet European regions are highly diverse, and we lack understanding of how different regional characteristics may enable or inhibit sustainability transitions. This hinders the development of context-tailored governance strategies. In this paper, we identify and apply sets of spatial indicators to map the regional potentials for agri-food transitions. We first analyse the strength of lock-in to the incumbent agro-industrial paradigm. We then map the enabling environments for two alternative agri-food networks—multifunctional value chains and civic food networks—that each embed distinct social–ecological qualities of agriculture and food. Results demonstrate a large spatial diversity in transition potential, with stronger lock-ins throughout North and Western Europe and stronger enabling environments for agri-food transitions in Italy, France, Switzerland, and Southwest Germany. We find that lock-ins are strongest in livestock-dominated regions and are associated with higher GHG emissions and excess nitrogen levels. Our study demonstrates the need for coordinated public policies that (1) leverage region-specific transition potentials and (2) enable complementary innovations in market-based and community-led networks.

**Supplementary Information:**

The online version contains supplementary material available at 10.1007/s11625-024-01480-y.

## Introduction

Today’s dominant, industrialised agri-food systems are not sustainable. There is consequently growing consensus that agriculture, and the wider agri-food systems in which it is embedded, must fundamentally transform towards arrangements that better serve both humanity and the planet (HLPE 2019; UN 2021; Willett et al. 2019). Although farmers are key actors for agricultural sustainability transitions, they do not exist in a vacuum, but are embedded in networks of actors who may promote or resist changes to the current paradigm (Williams et al. 2023). Transformative change therefore requires an enabling social, economic, and political environment in which actors can innovate to disrupt incumbent regimes (Blesh et al. 2023; Rutting et al. 2022). These enabling environments differ geographically (Coenen et al. 2012; Lamine and Marsden 2023), due to socio-political history and other regional factors, implying that fostering transformation requires context-specific policy and governance strategies (Bennett et al. 2021; Vermunt et al. 2020). Yet, we lack necessary understanding of *where* the greatest opportunities exist for transforming agri-food systems towards sustainability, and where systems are most locked-in into the dominant, industrial structure. This article addresses this challenge, focusing on Europe, which comprises diverse and geopolitically important agri-food systems facing many interacting stressors that may provoke—or hamper—transformation (Debonne et al. 2022).

Agri-food networks encompass the webs of actors, practices, and interactions involved in the production, processing, transport, consumption, and disposal or recycling of food (IPES-Food 2015), as well as underlying governance and knowledge systems. Networks include actors upstream and downstream from farmers in the value chain (VC), state actors at multiple scales, and a diverse range of third sector and civil society organisations (CSOs) (Avelino and Wittmayer 2016; Fischer and Newig 2016). The dominant kind of agri-food network in industrialised systems today is characterised by powerful VC actors, farmland concentration, globalised markets, commodification, and input-intensive agricultural practices (IPES-Food 2023). These networks are associated with many adverse sustainability outcomes (Frison and Clément 2020; Hendrickson et al. 2017; Wezel et al. 2018), with hegemonic actors and path-dependent processes that promote lock-ins of current development trajectories (Conti et al. 2021). Yet innovative farming practices and VC configurations are beginning to emerge within this inhibiting environment (Blesh et al. 2023; Poças Ribeiro et al. 2021), resulting in different kinds of agri-food networks that re-distribute power and re-value the social-ecological qualities of food (Renting et al. 2012; Rossi et al. 2019; Williams et al. 2023). These innovations are potentially precursors to regional sustainability transitions, wherein alternative (i.e. non-dominant) sets of practices and values gain dominance, either by replacing incumbent networks or through reorientations of regime actors (Geels and Schot 2007). The term ‘alternative’ is thus not relevant throughout all stages of a transition, but we use it here as an encompassing term to describe any kind of network (i.e. set of practices, values, and interactions) that departs from the currently ‘conventional’ industrialised paradigm.

Transitions to more sustainable agri-food networks emerge from the dynamic interplay of sets of enabling and inhibiting mechanisms. Factors that enable change include infrastructure for non-dominant VCs (e.g. farmers’ markets or food labelling schemes), financial or organisational support for innovative farming practices (e.g. agri-environment schemes or payments for ecosystem services), as well as supportive values, norms, and knowledge (e.g. consumer preferences for organic food) (Williams et al. 2023). Inhibiting factors, in contrast, include path-dependent processes such as increasing returns to adoption (e.g. technological development around dominant cereal crops (Magrini et al. 2016)) and tactics employed by powerful actors to maintain the status quo (e.g. corporations providing mis-information around pesticide health impacts (Hüesker and Lepenies 2022)), which can together generate lock-ins of current development trajectories (Conti et al. 2021).

A major gap in our understanding of lock-ins and enabling environments concerns how they vary across space. This spatial heterogeneity is likely to be significant, due to the global diversity of agri-food systems and development trajectories (Malek and Verburg 2020; Marshall et al. 2021). The lack of attention to the geography of agri-food transitions (El Bilali 2019; Lamine and Marsden 2023) is perhaps in part because the mechanistic knowledge on lock-ins and enabling environments is largely qualitative, having emerged from a rich body of case study research (Williams et al. 2023), and has thus far not been thoroughly integrated into large-scale quantitative analyses (though see Debonne et al. (2022)). Evidence on *where* different types of enabling environments are found—both those restricting and facilitating sustainability transitions—is a key input to agri-food transition governance, as it can help to tailor the necessary types and levels of external support (Oberlack et al. 2023; van Berkel and Verburg 2011); regions with stronger enabling environments are better poised for change, whereas weaker enabling environments require more directed policies and investment to overcome lock-ins.

These issues are particularly pertinent to European agri-food systems, where current policy mechanisms support agricultural practices that are among the most intensive in the world (Demay et al. 2023; Dietrich et al. 2012). State investment in European agriculture is high, and the recent Farm to Fork (F2F) Strategy (European Commission 2020) underscores the need for drastic food policy changes and integration to achieve the EU’s ambitious sustainability goals. Food policy integration could help to mitigate problems with the existing Common Agricultural Policy (CAP), which leans heavily on direct payments to farmers (i.e. underplaying the roles of other agri-food actors) and requires restructuring to strengthen sustainability (Linares Quero et al. 2022; Pe’er et al. 2019). The F2F and the 2023–2027 CAP present opportunities for context-specific implementation pathways (Guyomard et al. 2023) and empowering non-state actors (Moschitz et al. 2021). Europe has a large diversity of agri-food contexts (Jepsen et al. 2015; Mohr et al. 2023), so knowledge about the geography of European agri-food networks could facilitate appropriately contextualised action towards the policy objectives laid out in the European Green Deal and beyond.

**Fig. 1:**
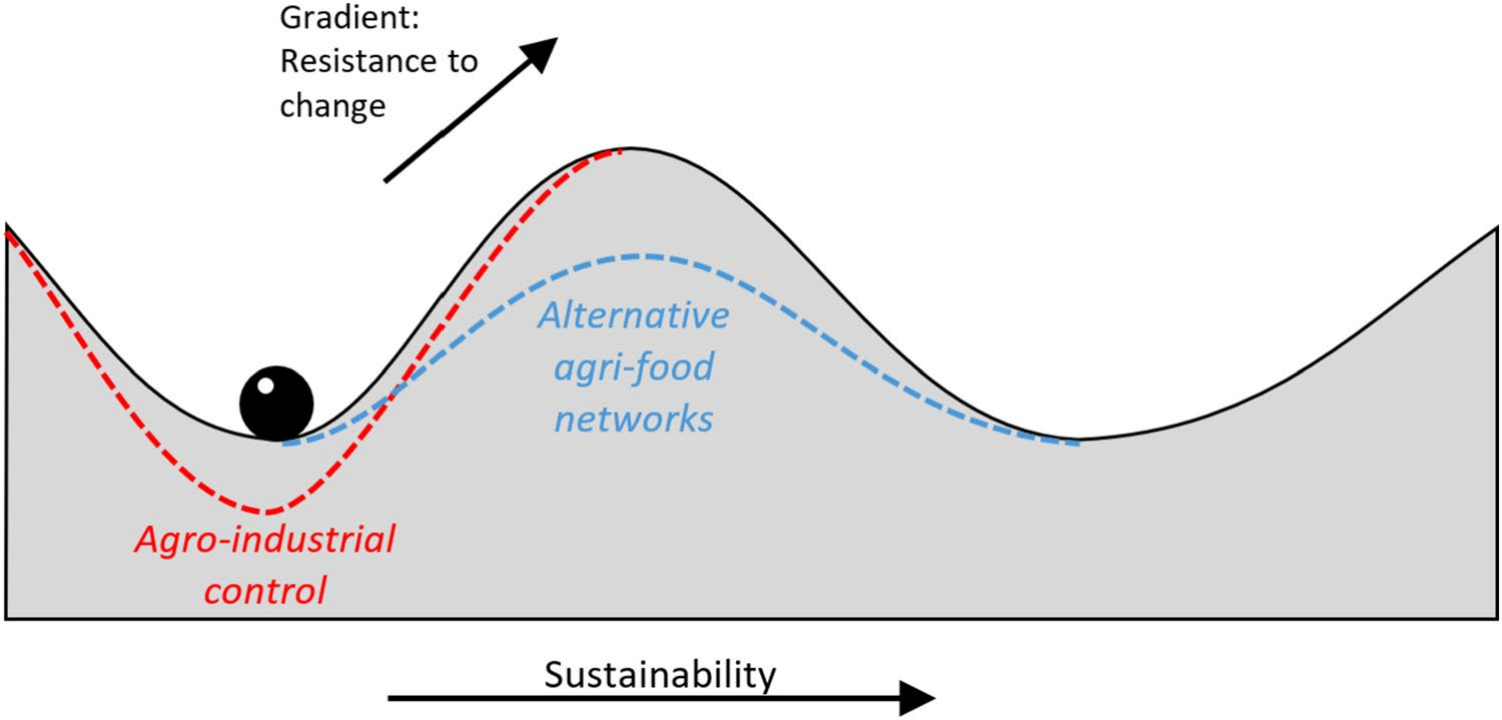
Schematic illustration of system stability [adapted from Gunderson (2000)]. Agro-industrial control contributes to lock-ins by inhibiting change, whereas alternative agri-food networks create enabling environments for sustainability transitions. This article maps these two features, and their coexistence, across European agri-food systems

This article maps the enabling environments for agri-food sustainability transitions in Europe (Fig. 1). We first analyse a set of spatial indicators that collectively represent the strength of lock-in of agro-industrial control. From this baseline, we then assess the overlapping enabling environments for two alternative agri-food networks: multifunctional VCs and civic food networks. Finally, we discuss how public policy might be tailored to regional contexts to foster transitions towards sustainability.

## Empirical and conceptual background

### Agri-food network types

Agri-food networks are based on the premise that farmers are embedded in social-material networks, and the power-laden interactions within these networks are intertwined with agricultural management, sustainability, and equity (Farstad et al. 2021; Gaitán-Cremaschi et al. 2019). For example, production for globalised VCs is more likely to use synthetic agro-chemicals and also, due to power exercised by transnational corporations, frequently disempowers producers (IPES-Food 2017). In relation to the broader concept of ‘agri-food systems’ (IPES-Food 2015), the term ‘agri-food networks’ in this study indicates the more specific focus on the actors and interactions within agri-food systems. Further, the term ‘agri-food’ (as opposed to, e.g. ‘food’ or ‘land’) reflects the motivation for understanding these networks to advance farming system sustainability (which may also produce non-food products such as animal feed, fibre, and biofuels), so each network includes the farmer. However, the network definitions do not describe the heterogeneity of farmers, which has been extensively characterised elsewhere (Bartkowski et al. 2022). Agri-food networks complement the more consumer-focused notion of ‘food environments’, which have been the subject of prior mapping studies (Lytle and Sokol 2017).

**Fig. 2:**
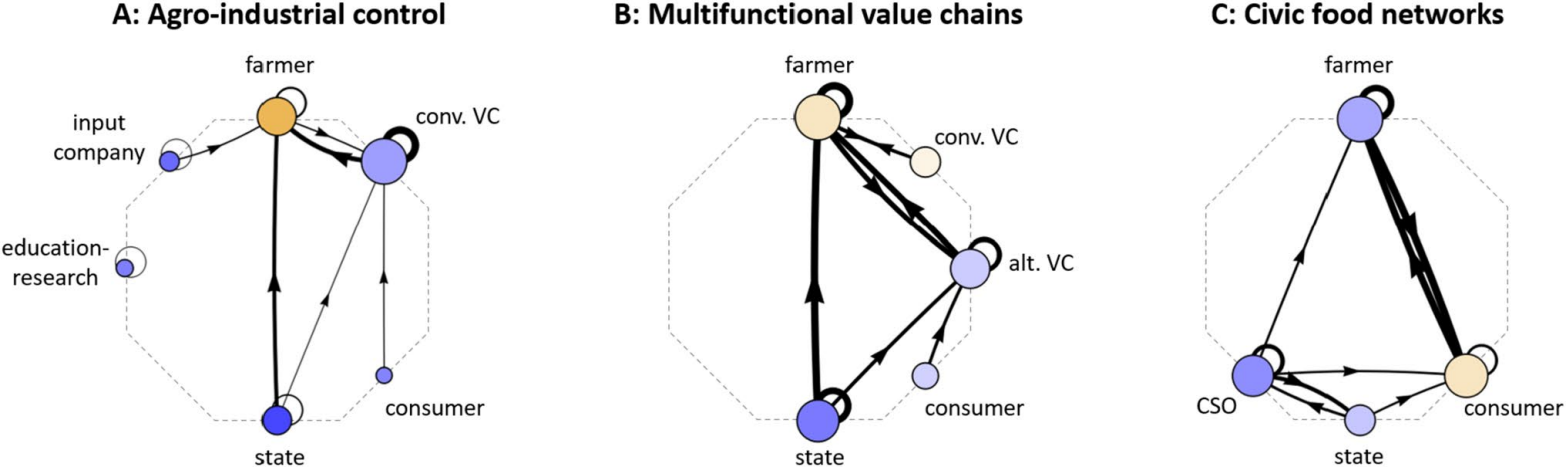
Representative network configurations for the three agri-food network types. The diagrams depict the actors and relationships most frequently discussed within narratives of these networks in the academic literature, based on Williams et al. (2023). The (eight) most frequently discussed actors are placed at consistent positions around an octagon to facilitate visual comparison. The size of the nodes and edges correspond to their relative importance. Orange shades indicate passivity and blue shades indicate activity

We consider three types of agri-food networks based on a semi-quantitative synthesis of European case study research (Williams et al. 2023) (Table 1; Fig. 2). The network typology categorises archetypical sets of actors and power-laden relationships that recur across diverse European contexts and correspond with different market configurations and levels of farmer autonomy. We conceptualise the first network configuration (type A) to currently dominate European agri-food systems, and types B and C as distinct alternatives to this dominant structure. The definitions of each network type are conceptually non-overlapping, but in reality hybrid networks exist (Lamine et al. 2012) and farmers may engage with multiple networks (e.g. by selling their products to both organic and conventional markets) (Brinkley 2017). Further, as the networks were built from case study descriptions, they reflect the systems studied in the academic literature and may not encompass all European contexts or actors.

**Table 1:** Characteristics of the three agri-food network types

	Network type		
	A: Agro-industrial control	B: Multifunctional value chains	C: Civic food networks
Description	Farmers are highly dependent on and influenced by VC and state actors	Organisational innovations in the formal VC place value on farmer autonomy, ecological, and/or regional food qualities	Farmers and consumers organise to create relations outside of mainstream markets
Guiding logic	Food as a commodity	Food value includes social-ecological qualities	Food as a community good
Defining features	State regulation Subsidies Advisory services R&D Contract farming Private quality standards	Collaborative relationships Innovation Sustainability labels/brands State funding and support Peer influence Capacity building	Actors unite CSOs lobby the state Knowledge sharing Personal relationships Direct trade Changing attitudes and values
Dominant governance mode (Pahl-Wostl 2015)	Hierarchical	Market	Network
Loci of power	State; conventional VCs; input companies	VCs (conventional and alternative); state; farmer; consumer	Farmer; consumer; CSOs
Market configuration	Large-scale industrialised VCs	Large-scale values-based VCs; local VCs	Direct sale; local aggregation
Relative farmer autonomy	Low	Medium	High
Power is exercised by	Organisations	Organisations	Individuals and organisations

Network type A, *agro-industrial control*, is the dominant configuration in narratives of current European agri-food systems (Frison and Clément 2020; Jepsen et al. 2015; Levidow 2015) to the extent that “corporate influence…has become the new normal” (IPES-Food 2023, p. 4). VC actors have strong influence over these networks, for instance through retailers (i.e. supermarkets) setting prices and creating contracts that incentivise or require specific farming practices and/or quality standards (Dewick and Foster 2018; Dries et al. 2004; Rossi et al. 2019), or the seed and chemical sector using public relations to shape narratives about agriculture (Clapp 2021). In this network type, the central objective of agricultural management is to maximise productivity and/or efficiency, often involving intensive use of external inputs (e.g. fertiliser, pesticides, energy), reduced labour, and low farm-level and regional diversity of agricultural products. State actors (and corporate actors who influence them) play strong roles in regulating environmental impacts, funding research, and providing production-oriented subsidies and advice to farmers (Kuokkanen et al. 2017; Vanloqueren and Baret 2008). Environmental considerations may play some role in these networks (i.e. a “corporate–environmental food regime” (Friedmann 2005)), but the networks still reinforce a productivist narrative (Levidow 2015). While it is difficult to quantify the dominance of agro-industrial control, many statistics point to the overwhelmingly industrialised state of European agri-food systems, with, for example, farmers’ input costs (e.g. for seeds, fertilisers, antibiotics) comprising 41% of their total revenue on average (European Commission 2019a) and the four largest supermarket firms controlling 60% of the retail market in the average European country (Van Dam et al. 2021).

Network type B, *multifunctional value chains*, represents an alternative to agro-industrial control wherein non-economic qualities of farming and food products are integrated into formal VCs. This can involve collaborative relationships with conventional VC actors that increase farmer autonomy (De Herde et al. 2020), as well as trade through novel VCs involving actors such as small-scale cooperatives, local stores, or food hubs (Poças Ribeiro et al. 2021). Multifunctional VCs can embody a range of non-economic values, such as short supply chains (Chiffoleau and Dourian 2020), territorial embeddedness (Flinzberger et al. 2022), or ecological and/or animal-friendly farm management (Renting et al. 2003). These values are integrated into commodity markets, often through formal certification schemes and labels, such as organic agriculture or Protected Designation of Origin (PDO). As the associated agricultural and marketing practices often require new knowledge, technology, and infrastructure, these networks are frequently supported by state funding (e.g. agri-environment schemes within the CAP) (Williams et al. 2023). Multifunctional VCs are growing but still marginal in Europe on both the supply and demand sides, with organic agriculture (i.e. supply) covering only 9.9% of the total EU agricultural land in 2021 (Austria is highest with more than 25%) (Eurostat 2023) and the organic retail market (i.e. demand) comprising less than 10% of most national markets (Denmark has the global highest, with 12.1% in 2019) (Willer et al. 2021). Products with registered geographical indications represented 7% of total EU food and beverage sales in 2017 (European Commission 2019b).

Network type C, *civic food networks*, represents a second alternative to agro-industrial control wherein civil society actors such as citizen-consumers, farmers, and CSOs actively create and govern food systems (Renting et al. 2012). In these networks, formal VCs and state actors play less prominent roles and most power is exercised through bottom-up forms of organisation (Williams et al. 2023). Farm products are directly sold to consumers, for instance through farm shops, farmers’ markets, or community-supported agriculture (CSA) schemes in which citizen-consumers play an active role in the management and even production of the food (Hinrichs 2000). Within these networks, consumer–producer relationships expand from being purely economic to encompass social and political aspects, such as community and solidarity (Renting et al. 2012). CSOs can play important roles by connecting different types of actors, providing education, and lobbying state actors for policy change (van Gameren et al. 2015). Civic food networks are also growing but marginal in Europe, with, for example, 474,000 Europeans participating in CSA schemes (as of 2015) (Urgenci 2016), which is less than 1% of the population.

We interpret the network configurations and their implications for sustainability with an intentional and normative directionality. By sustainability we refer to the contribution of agri-food systems to the stability and resilience of the earth system and human wellbeing, including justice considerations for current and future generations (Gupta et al. 2023). Power asymmetries represent a form of injustice, and also interact as drivers and outcomes of other sustainability dimensions (Leach et al. 2018). Specifically, we interpret network type A as the hegemonic configuration that, by definition, affords farmers less autonomy and treats the environmental effects of agriculture as externalities. Besides the injustice of the asymmetric power balance, ample evidence demonstrates that such approaches have insufficiently improved sustainability outcomes (Béné, 2022; Chaplin-Kramer et al. 2022; Scown et al. 2020). It is indeed possible that corporate activities can align with sustainability (Österblom et al. 2022), but risks of elite capture arise without intentional and democratic inclusion of other actors (Leach et al. 2018). Network types B and C contrast with this regime as they promote procedural and distributional justice through more balanced power distributions and lower levels of farmer dependence (Williams et al. 2023). By embedding the social-ecological qualities of food, they also have the potential to generate more holistic and beneficial sustainability outcomes (Hendrickson 2015; van der Ploeg et al. 2019). Nevertheless, trade-offs will inevitably arise under any configuration (Chiffoleau and Dourian 2020), so we cannot claim that any network is unilaterally preferable, only that networks B and C offer greater potential for human and planetary well-being.

### Mechanisms promoting lock-in and transitions to sustainability

Agri-food networks are in reality dynamically evolving entities that are shaped by a complex interplay of internal processes and external forces. These processes and forces can both consolidate and undermine existing network structures over time. To make progress towards more sustainable agri-food networks therefore requires addressing the mechanisms that promote lock-in and facilite transitions in actors’ values and practices.

Lock-ins are path-dependent processes caused by combinations of self-reinforcing mechanisms that together inhibit change on a systemic level. Self-reinforcing mechanisms in agri-food systems include various forms of increasing returns to adoption (Arthur 1989), whereby technology, infrastructure, and knowledge develop around the practice that is initially adopted (e.g. cereal crops or genetic engineering) (Magrini et al. 2016; Meynard et al. 2018; Vanloqueren and Baret 2008). Even if this practice is inferior to other alternatives, its relative performance will increase over time (Cowan and Gunby 1996). In conjunction with sunk costs of investments and aversions to risk and loss (Kahneman and Tversky 1979), it becomes increasingly difficult to change the socio-technical paradigm (Vanloqueren and Baret 2009). But systemic resistance to change is often more than inertia; the status quo is also preserved through conscious efforts by highly capitalised actors (Seto et al. 2016), who exercise power over others (Partzsch 2017) through tactics such as lobbying and spreading of mis-information to influence public opinion and policy decisions (e.g. by downplaying the risks of pesticides to human health) (Chambers 2016; Hüesker and Lepenies 2022). State interventions, and the underlying knowledge supporting those interventions, also usually reinforce orthodox ideologies based on economic growth (Benton 2023), making it difficult for more transformative solutions to gain traction. These socio-political processes dynamically interact with technological and behavioural mechanisms, across scales, to produce deep lock-ins in agri-food systems (Conti et al. 2021; Oliver et al. 2018).

Transitions are also path-dependent processes caused by combinations of self-reinforcing mechanisms (Anderson et al. 2019), but these mechanisms work to *disrupt* the incumbent regime. The mechanisms that facilitate sustainability transitions are thus somewhat similar to the lock-in mechanisms described above, but differ in the kinds of practices and values they promote as well as in the way they act to oppose dominant structures. Two necessary initiating factors for transitions are therefore values that stand in opposition to existing paradigms (Martin et al. 2022) and, relatedly, a degree of frustration with current conditions (e.g. farmer marginalisation, health-related shocks, soil degradation) (Mier y Terán Giménez Cacho et al. 2018; Rossi et al. 2019). Supportive institutional environments [e.g. governance, markets, and norms (Bacon et al. 2012)] can help provide the necessary resources for frustrated actors to change their practices and build alternative networks (Blesh et al. 2023; Williams et al. 2023). Here, power is collectively exercised with others as well as by individuals to accomplish their goals (Partzsch 2017). For transitions to succeed requires cooperation across the entire VC, including farmer innovation, consumer demand, and infrastructure to bring products from farm to market (Gava et al. 2022). Existing alternative networks therefore also provide a foundation—of practices, values, and interactions—from which further innovations can precipitate, for instance as niche actors institutionalise their practices and influence others’ values (Bui 2021).

It follows that the mechanisms promoting lock-in and transition also interact with—and oppose—each other. In other words, the lock-in mechanisms can be considered as *disabling* factors that suppress transitions, and vice versa (Anderson et al. 2019). In our context, lock-ins to agro-industrial control thus constitute a form of undesirable resilience (Oliver et al. 2018), enabling environments for multifunctional VCs and civic food networks represent transformative capacity (Béné et al. 2012), and a region’s transition potential emerges from the interplay of these two sets of opposing forces. Our spatial analysis of these system features is particularly novel, as the mechanisms described above are typically analysed through case studies and discussed in qualitative formats (Conti et al. 2021), with the *spatiality* of lock-ins and transitions being rarely addressed (El Bilali 2019; Köhler et al. 2019) [for an exception, see Debonne et al. (2022)].

## Data and methods

### Spatial indicators

Although the agri-food networks describe distinct configurations of actors, practices, and values, they are difficult to measure directly, i.e. the networks are *latent constructs* (Byrne 2005). This is because a network type cannot be completely described through a single observable behaviour, and actors’ underlying values and power relations are difficult to quantify. To overcome this challenge, we develop a set of spatial indicators that collectively represent the enabling environment for each agri-food network type (Table 2). Each indicator represents either a condition supporting the emergence of the network (e.g. physical or institutional infrastructure) or signs of the network’s existence (e.g. network outcomes or proxies for the relations between actors). As we conceptualise agro-industrial control as the baseline (undesirable) network configuration, the indicators that describe its enabling environment collectively represent the strength of lock-in.

**Table 2:** Indicators for the enabling environments of each agri-food network type at the NUTS 2 spatial level

	Indicator	Interpretation	Calculation details	Year(s)	Data source(s)
Type A: Agro-industrial control					
1	External input intensity	Farmers’ dependence on input companies	€ inputs (crop and livestock)/€ agricultural production Inputs for crops include seeds and plants, fertilisers, crop protection, and other specific costs. Inputs for livestock include feed, veterinary costs, and other specific costs	2016–2020 average	FADN
2	Indebtedness	Farmers’ dependence on banks and sunk costs of investments	Total liabilities/farm net income	2016–2020 average	FADN
3	Value chain concentration	Power of value chain actors	Average of three national-level metrics, representing economic concentration in (a) manufacture of food products, (b) wholesale of agricultural raw materials and live animals, and (c) supermarket industry	(a) 2020, (b) 2020, (c) 2017	Eurostat (Van Dam et al. 2021)
4	Subsidy intensity	Farmers’ dependence on state subsidies	€ CAP Pillar I payments/€ total agricultural economy	2015 (CAP); 2015–2020 (Eurostat)	Nicholas et al. (2021); Eurostat
5	Labour productivity	Scale enlargement of farms. See Van der Ploeg et al. (2019) for a detailed discussion of its links to industrial agriculture	€ agricultural output (i.e. revenue) per unit of labour	2015–2020 average (revenue); 2016 (labour)	Eurostat
6	Low production diversity	Regional dependence on few output markets	Concentration of regional production across product categories (e.g. dairy, wine, fresh vegetables, grain maize). Measured using the Herfindahl–Hirschman Index (HHI)	2015–2020 average	Eurostat
Type B: Multifunctional value chains					
1	Organic farming	Farmer innovation and value shifts	Fraction of farmers who are organic (fully converted or under conversion)	2020	Eurostat
2	Protected designation of origin (PDO)	Opportunity for values-based market differentiation	Count of registered food products	2020	Flinzberger et al. (2022)
3	Consumer perceived organic access	Evidence of developed organic VCs	Fraction of survey respondents who do not find it difficult to find organic food products in their area	2022	EU Barometer
4	State-led rural development	State support for farmer and rural innovation	€ CAP Pillar II payments/€ total agricultural economy	2015 (CAP); 2015–2020 (Eurostat)	Nicholas et al. (2021); Eurostat
5	Agricultural innovations	Farmer innovation and collaboration with other actors (e.g. researchers, NGOs)	Count of EIP-AGRI Operational Groups, normalised by the number of farms	2022	European Commission; Eurostat
6	Farmer cooperatives	Improved bargaining power and reduced risk for farmers	Percentage of farmers who are members of a cooperative (excluding dairy)	2010	Bijman et al. (2012)
Type C: Civic food networks					
1	Farm shops and vending machines	Farmer innovation and direct producer–consumer interaction	Number of farm shops and vending machines divided by the total number of mapped amenities	2022	OpenStreetMap
2	Farmers’ markets	Direct producer–consumer interaction in values-based food networks	Number of marketplaces divided by the total number of mapped amenities	2022	OpenStreetMap
3	Consumer willingness towards alternative food	Consumer interest in values-based food systems	Fraction of survey respondents who agree that: (a) it is important that their food comes from a geographical area they know; (b) it is important that their food respects local tradition and know-how; and (c) they are prepared to pay 10% extra for products with lower carbon footprints. Average of the three values	2022	EU Barometer
4	Community-supported agriculture (CSA)	Solidarity and relationship-based trade between farmers and citizen-consumers	Number of CSA consumers, normalised by the regional population	2015	Urgenci (2016)

Further calculation details and data citations are given in Supplementary Material A

To ensure that each indicator set sufficiently proxies its agri-food network, the main requirement when developing the indicators was that they collectively describe the practices and/or values of *multiple* agri-food actors (e.g. not only farmers). We first created an initial set of indicators based on the recurrent processes observed in empirical case study research (Williams et al. 2023). We then circulated a questionnaire to 18 regional academic experts to verify the extent to which the analysis accurately represented their understanding of regional agri-food systems, and used their responses to refine the indicator set and calculation approaches. There were ten responses to the questionnaire, with expertise in 13 European countries [see Supplementary Material (SM) A]. This process resulted in six indicators for type A networks, six for type B, and four for type C (Table 2). The indicators collectively describe farming practices (A1, A6, B1), VC infrastructure (B2, B3, B6, C1, C2, C4), consumer values (C3), policy environments (A4, B4, B5), and network outcomes (A2, A3, A5).

All analysis is conducted at the NUTS2 level (Nomenclature of Territorial Units for Statistics, i.e. the territorial units for the framing and definition of EU regional policies) and spans the EU27 plus the United Kingdom (UK) and Switzerland. For all indicators, we sought the most recent available data. Calculation details are included in SM A and the final dataset is provided in the Online Supplement.

Due to the limited availability of Europe-wide data, the indicators do not always capture all aspects of the theoretical network type. This is particularly the case for network type C. For instance, many forms of direct marketing exist (Poças Ribeiro et al. 2021) that are difficult to measure at a European level, and there is no centralised or public data on farmers’ contract conditions or the activities of CSOs. The analysis therefore cannot be used to make *absolute* comparisons between network types, i.e. we cannot infer which type is dominant or stronger in a particular location. Rather, the spatial indicators combine to explain how the *relative* strength of evidence varies across Europe. Even so, this approach is imperfect and for instance misses national and regional policies as well as informal practices (e.g. farmers practising organically but not formally registered). This may lead to geographic biases, as some agri-food systems may rely more heavily on informal institutions (Visser et al. 2015). Also, OpenStreetMap (for indicators C1 and C2) has better coverage in some areas than others; our strategy to mitigate this bias is described in SM A. We interpret our results in the light of these considerations.

### Analysis

#### Enabling environments

For each network type, we integrate all indicators to create an “index” between 0 and 1. The index for network type A represents the relative strength of lock-in to agro-industrial control, whereas the indexes for types B and C represent the enabling environments for different kinds of sustainability transitions. We first normalise each indicator to a consistent [0,1] range by calculating quantiles. Thus, the NUTS2 region with the lowest value for a given indicator receives a value of zero, the median receives a value of 0.5, and the maximum receives a value of one. Then, for each NUTS2 region, we calculate the average quantile across all indicators within each network type, with all indicators receiving an equal weight. Finally, we calculate quantiles across each index to again yield values uniformly distributed across the [0,1] interval. This process effectively calculates a combined weight of evidence for each network type, which enables identifying regions that score relatively high or low across all component indicators. Regions that have diverging indicator values (e.g. high subsidy intensity (A4) but low input intensity (A1)) will score moderately in the combined index. Given our focus on agriculture, we exclude NUTS2 regions with less than 10% agricultural area (*n* = 5) as well as city areas (*n* = 12), which may indeed be sites for civic food networks but do not contain necessary agricultural data for several variables. When calculating each index, we retain regions with missing data for one or two layers. The final dataset includes 263 NUTS2 regions (excluded regions are listed in SM A).

To assess the robustness and trustworthiness of our results, we conduct two sensitivity analyses and one validation. First, to examine the sensitivity to the standardisation process, we re-calculate the indexes using a min–max scaling instead of the quantile approach described above. The min–max scaling better preserves the absolute differences between data values, but is more sensitive to outliers as it maintains the original data distribution. The second sensitivity analysis examines the sensitivity to the choice of the indicators, by iteratively excluding and doubling the weight of each indicator in the index calculations (Prestele et al. 2018). For both sensitivity analyses, we examine their effects on the spatial patterns of the resulting indexes (see SM C). For the validation, we examine how the mapped indexes align with an independent dataset of spatially locatable empirical case studies of agri-food networks [*n* = 26 national-level and *n* = 38 local/regional case studies, based on Williams et al. (2023)]. This comparison is only indicative, as the case studies are not necessarily representative of their wider regions, but they provide the best available independent reference against which to compare our results.

As each index is calculated separately, a single region may score high (or low) for multiple indexes. This is due to the possible coexistence (or absence) of multiple network configurations within farms or landscapes, as well as the diversity of landscapes within a mapped NUTS2 unit. Thus, in addition to assessing the spatial distribution of each individual network type, we also overlay the indexes to identify regions with: (i) high transition potential (weak A, strong B and C), (ii) competing networks (strong A, B, and C), (iii) strong lock-in (strong A, weak B and C), and (iv) limited evidence (weak A, B, and C). Overlaying the indexes in this way helps examine their spatial alignment to understand regional potentials for agri-food transitions.

#### Assessing associations with location factors

Agri-food networks are intertwined with sustainability and development; certain types of networks may be more likely to emerge in particular socio-economic or environmental contexts, and they may generate different rural development pathways and sustainability outcomes. We conduct two sets of correlation analyses to examine how the enabling environments overlap spatially with a range of societal indicators (Table 3). The first analysis focuses on contextual factors that are often used to characterise farming systems (Bartkowski et al. 2022; Guarín et al. 2020; Weltin et al. 2018), comprising variables that relate both directly to the agricultural system (crop suitability, farmer age, livestock prevalence) and to the socioeconomic context in which farming is embedded (population density, GDP). The second analysis focuses on a set of megatrends facing agriculture, including environmental policy gaps (Debonne et al. 2022), risks due to climate change, risk of land abandonment (Perpiña Castillo et al. 2021), and water stress (Hofste et al. 2019). These megatrends place spatially divergent pressures on agri-food systems that could strengthen lock-ins or lead to systemic change (Debonne et al. 2022), and this analysis helps to understand the policy pressures and risks that different kinds of agri-food networks face. SM A provides data sources and calculation details.

**Table 3:** Analyses for testing the associations between the agri-food networks and other relevant location factors

Analysis type	Example questions	Location factors
Contextual factors	Do we see stronger *agro-industrial control* in regions with dominant livestock economies? Are regions with stronger *civic food networks* more affluent (i.e. higher GDP)?	Population density GDP per capita Crop suitability Farmer age ratio (number of farmers ≥ 55 per farmer < 55) Livestock (fraction of agricultural economy)
Megatrends	Are agricultural GHG emissions higher in regions with high *agro-industrial control*? Do regions with strong *multifunctional value chains* face higher risks of land abandonment?	Environmental policy gaps (Agricultural GHG emissions per hectare; Excess nitrogen) Other risks (Climate change [decreasing yield or increasing drought risk]; Land abandonment; Water stress)

We calculated the Spearman correlation coefficient between each network index and location factor

#### Tabulating policy instruments

State actors play central roles in setting the policy environments for European agri-food systems (Soriano et al. 2023; Williams et al. 2023). The indicators related to the CAP (A4 and B4 in Table 2) are broadly indicative of how state spending differs across Europe (Nicholas et al. 2021) and can provide enabling environments for different network types. For instance, if area-based payments (from CAP Pillar I) comprise a large fraction of the regional agricultural economy, it suggests that farming is dependent on this money, and farmer dependence is a key characteristic of network type A. Agri-environment schemes in CAP’s Pillar II may also facilitate a degree of farmer dependence, but these payments support multifunctional agriculture and enable farmer innovation, which are characteristic of network type B. Beyond and within these two generic pillars, state actors have many other instruments at their disposal that cannot be easily summarised in European-level spatial datasets, so here we examine how a range of public policy instruments align with the three network types. For this exploratory, qualitative assessment, we draw relevant policy instruments from the synthesis of Galli et al. (2020) and classify the instruments, based on the actors and/or interactions that they target, as helping to strengthen one of the three agri-food network types. For simplicity, we focus on actions available to state actors, but discuss the results from the perspective that other actors also play important roles in food system governance.

## Results and discussion

### Spatial distributions of the enabling environments

#### Agro-industrial control

**Fig. 3:**
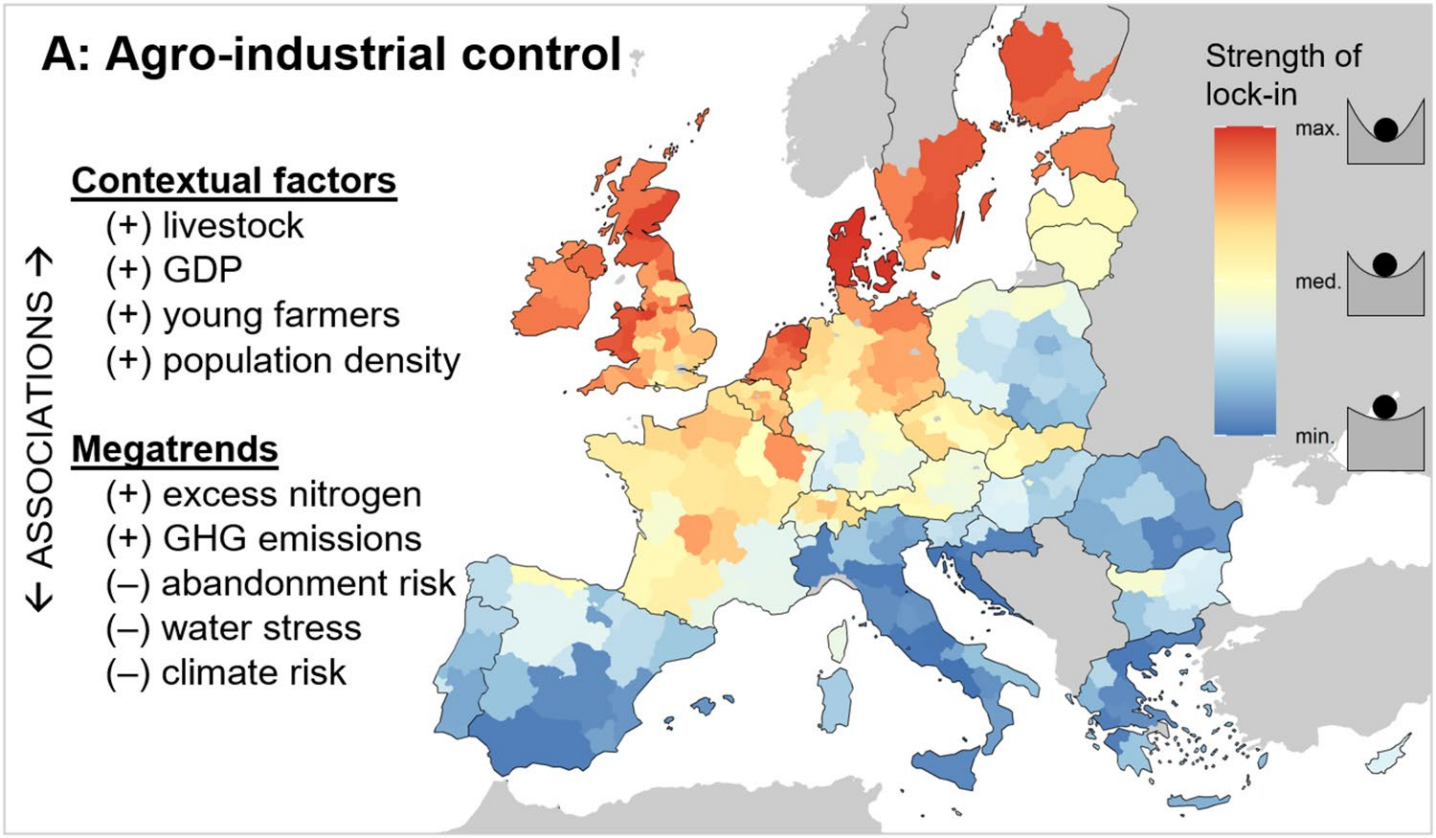
Profile of the *agro-industrial control* network type, showing the regional variation in the strength of lock-in and its associations with location factors. The + /– symbols denote positive/negative correlations (*p* < 0.05). Individual indicator maps and the quantitative statistical results are shown in Supplementary Material B. See Table 2 for the list of indicators comprising *agro-industrial control* and Table 3 for the definitions of the location factors

We found a clear north–south gradient of agro-industrial control in Europe (Fig. 3), with strongest evidence of lock-in in the north (particularly Scandinavia, Netherlands, and the UK) and weaker evidence in the south (particularly Italy, Greece, Croatia, Romania, and Spain). This pattern aligns with other descriptions of European agricultural diversity, characterised by more intensive land management in Central and Western Europe (Levers et al. 2018) and a higher proportion of traditional, less-industrialised agricultural livelihoods in parts of Eastern and Southern Europe (Jepsen et al. 2015). Some countries show considerable sub-national heterogeneity, in particular Germany, with higher agro-industrial control in the east—a region with a history of state-run collectivised farms followed by a transition to corporate land management after its reunification (Wolz 2013). The spatial results are robust to the choice of indicators and scaling method, and align well with the empirical case studies from a prior analysis using this typology (SM C). Nevertheless, some well-known intensive agricultural landscapes do not stand out in these results, such as the Po Valley in northern Italy and Almería in southern Spain (Rega et al. 2020). This is potentially because our agricultural intensity indicator (A1 in Table 2) represents the cost of inputs relative to the economic value of agricultural production, and these regions are intensive but also economically productive. The NUTS2-level analysis also obscures sub-regional heterogeneity, thus impacting the results in regions with heterogeneous agricultural systems.

The components of agro-industrial control often co-occur, leading to strong regional signals and clear spatial gradients. Several of the sub-indicators are correlated (Fig. B4), for instance external input intensity is positively associated with debt, labour productivity, and VC concentration. Agro-industrial control is significantly associated with all contextual factors except crop suitability (Table B1), with, for instance, stronger lock-ins in regions with dominant livestock sectors. The stronger agro-industrial control in livestock-dominated regions agrees with recent descriptions of concentrated animal feeding operations as “a hallmark of industrial agriculture” (Debonne et al. 2022, p. 8), which are particularly prevalent in Denmark, the Netherlands, and Belgium.

We found that regions with strong agro-industrial lock-in have statistically higher excess nitrogen levels and agricultural GHG emissions per hectare, underlining the imperative to improve sustainability in such regions (Table B2). These regions face relatively low risks of land abandonment, climate change, and water stress [a result of historical intensification in agriculturally favourable areas (Levers et al. 2018)], suggesting that the importance of agriculture in these regions is unlikely to decrease and environmental policy must contend with the actors and processes that contribute to lock-in of these networks. But as powerful, vested interests (both the agro-industry and their allies in the farming community) fight to defend the status quo (van der Ploeg 2020), it may be difficult to make meaningful change to mitigate these environmental problems within these networks (Béné, 2022).

These results imply that market actors (e.g. input companies, wholesalers, retailers) exert comparatively more control over agri-food systems in Northern Europe. Farmers’ and consumers’ autonomy is consequently relatively low. Other evidence corroborates this finding. We find that Denmark has the strongest evidence of lock-in, and other analyses have revealed the high indebtedness and vulnerability of large-scale Danish dairy farmers (van der Ploeg et al. 2019). In the UK, retailer concentration and the proliferation of ‘home brands’ have been linked to “(cartel-like) economic buying power” (Richards et al. 2013, p. 240) in which farmers are “in the hands of the retailer”. In Finland, farmers’ dependence is not restricted to economic realms (with subsidies comprising 40% of their incomes in 2014); intensification has also resulted in losses to localised knowledge, thereby increasing farmers’ dependence on authorities and advisors from the agro-industry (Kuokkanen et al. 2017). Historical dismantling of public extension services has contributed to these issues (Vanloqueren and Baret 2008). Ultimately, these dependencies and lack of diversity can lead to considerable risks to farmers’ socio-economic wellbeing (Helfenstein et al. 2022), and there is evidence that high levels of decoupled income support (from CAP Pillar I) can compromise the resilience of agricultural incomes in the face of shocks (Slijper et al. 2021). Despite the non-discriminatory design of these payments (i.e. all farmers can receive them if they comply with some basic conditions), our data suggests that these payments have highly variable levels of importance for different regions’ agricultural economies (mean 14%; SD 9%).

The relatively weak evidence of lock-in in Southern and Eastern Europe does not necessarily imply that agro-industrial control is *low* in these places. European agriculture as a whole is strongly influenced by the CAP, which largely supports intensive agriculture (Pe’er et al. 2020). Since 1970, all European regions have at some time been characterised by industrialisation, and “high-input, intensive agriculture continues to dominate” (Jepsen et al. 2015, p. 60) even with spreading environmental awareness. Nevertheless, the level of industrialisation differs geographically and traditional, subsistence-oriented agricultural livelihoods remain prevalent in some places (Varga 2019). Agro-industrial control may therefore be low in absolute terms in these regions, but throughout this paper we continue to interpret the results in a relative sense.

#### Multifunctional value chains

**Fig. 4:**
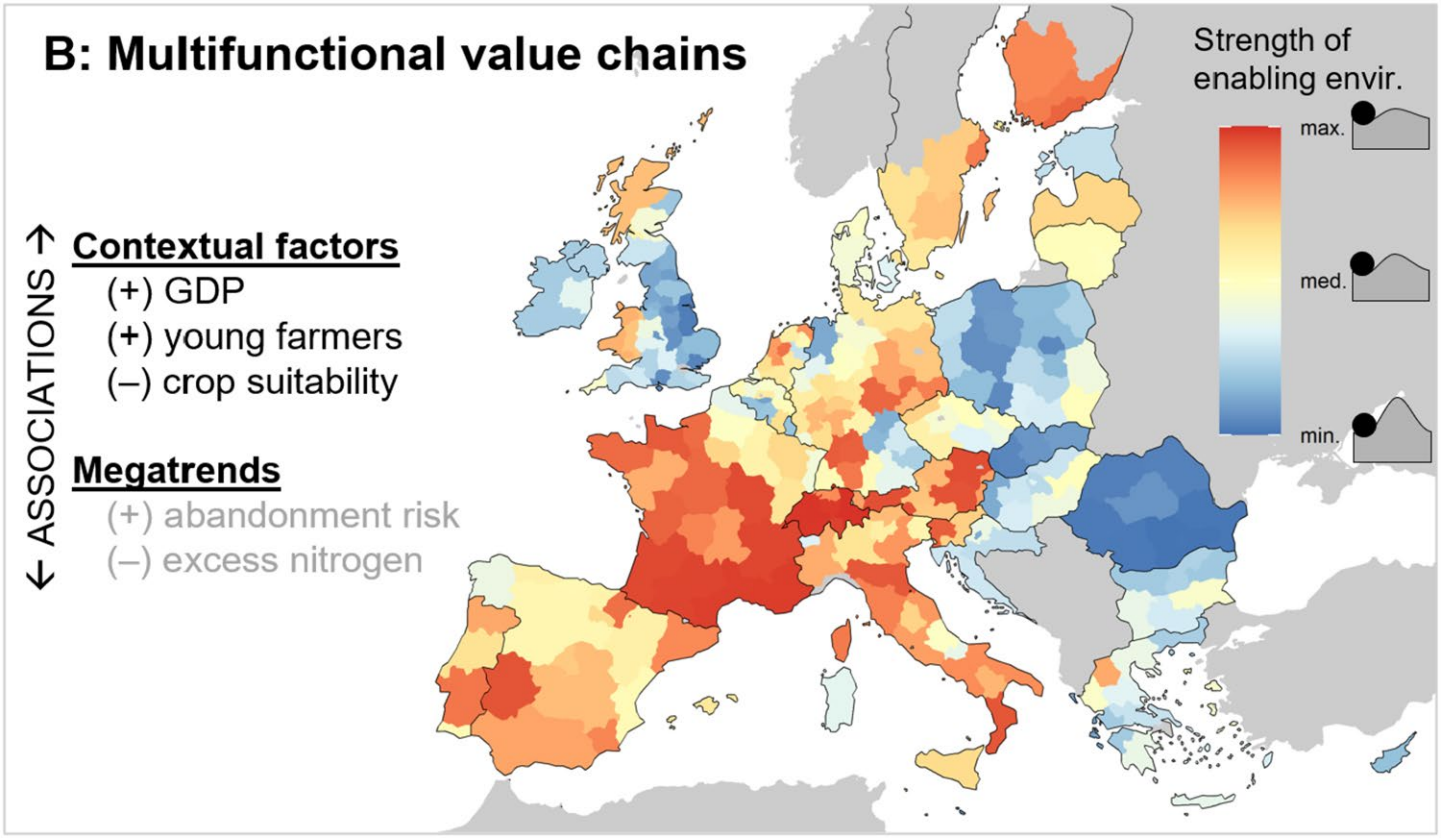
Profile of the *multifunctional value chains* network type, showing the regional variation in enabling environments and their associations with location factors. The +/– symbols denote positive/negative correlations (black text *p* < 0.05, grey text *p* < 0.1). Individual indicator maps and the quantitative statistical results are shown in Supplementary Material B. See Table 2 for the list of indicators comprising *multifunctional value chains* and Table 3 for the definitions of the location factors

Multifunctional VCs have a markedly different spatial signature to agro-industrial control; their enabling environments are generally strongest in mountainous regions, France, and Italy (as well as Eastern Germany, Finland, and Portugal), and weakest in Eastern Europe, the UK, and Ireland (Fig. 4). These patterns make sense in light of European cultural and agrarian histories; many regions have strong histories of (agri)cultural heritage being tied to landscapes (Plieninger et al. 2006), which has persisted to a greater extent in mountainous and marginal landscapes where agricultural modernisation was less feasible. Such cultural heritage is now often formally supported by EU rural development programmes and recognised through international labels such as PDO (Flinzberger et al. 2022). The results are generally robust to the indicator selection and scaling approach, but Switzerland has a moderate sensitivity due to missing data for two indicators (Fig. C1). The index values tend to be high in documented local case studies of multifunctional VCs (SM C).

The low values in Eastern Europe may indeed reflect weaker enabling environments for multifunctional VCs, but also demonstrate the limitations of a European-scale analysis. These results may be explained by less established networks of cooperation following the fall of the Soviet Union and/or poor targeting and implementation of EU rural development initiatives (Fieldsend et al. 2021; Mikulcak et al. 2013). However, some countries have national systems for registering traditional food products that are not captured by the EU-level dataset (Bichescu and Stanciu 2017). Production and consumption practices in these regions may also embody the values of multifunctional VCs without formal integration into national policy frameworks or datasets. Interestingly, the former Eastern Germany scores relatively high compared to the other post-socialist countries, which is due to higher prevalences of organic farming, EIP-AGRI operational groups, and farmer cooperative membership (SM B).

The sub-indicators are less strongly correlated than for agro-industrial control (Fig. B5), resulting in a fuzzier spatial pattern and fewer significant correlations with location factors (Tables B1; B2). The enabling environments for multifunctional VCs are stronger in wealthy regions and regions with higher proportions of young farmers. We also find that arable crop suitability is lower in regions with relatively strong multifunctional VCs, and these regions also face higher risks of land abandonment. This correlation is relatively weak, however, and we also observe relatively strong multifunctional VCs in some non-marginal production regions, such as parts of the Netherlands and East Germany, which is primarily driven by large numbers of agricultural innovations (B5 in Table 2).

The results imply that farmers in mountainous regions, France, and Italy have relatively more collaborative relationships and are better able to generate value through distinctive regional characteristics and ecological farm management. This agrees with a recent study on agricultural development pathways that found more multifunctional development in mountainous or otherwise marginal regions, especially in high-income countries (Helfenstein et al. 2023). This is potentially because these forms of product differentiation have emerged as strategies to mitigate abandonment risks (Flinzberger et al. 2022), and European governments explicitly support farming in marginal landscapes, e.g. through Pillar II of the CAP. It is difficult to make concrete conclusions about farmer autonomy in such situations, as farmers’ economic viability can become dependent on payments to offset the higher production costs in marginal regions or under organic agriculture (Jitea and Arion 2015). There is, however, evidence showing that agri-environment payments (and the associated agricultural diversity) can increase income stability (Harkness et al. 2021; Slijper et al. 2021).

#### Civic food networks

**Fig. 5:**
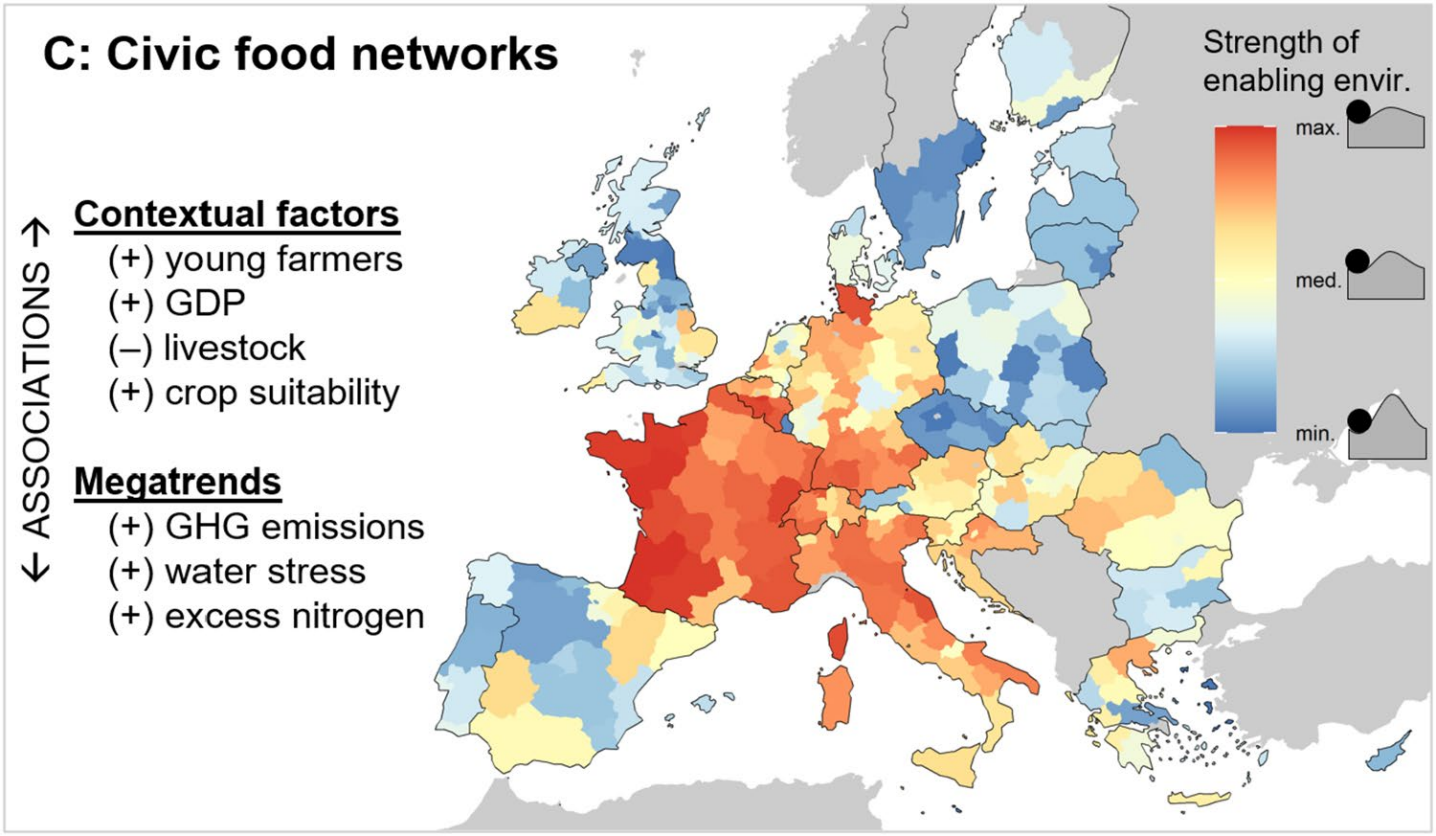
Profile of the *civic food networks* network type, showing the regional variation in enabling environments and their associations with location factors. The +/– symbols denote positive/negative correlations (*p* < 0.05). Individual indicator maps and the quantitative statistical results are shown in Supplementary Material B. See Table 2 for the list of indicators comprising *civic food networks* and Table 3 for the definitions of the location factors

Enabling environments for civic food networks are strongest in France, Belgium (particularly Wallonia), Switzerland, and Italy, as well as parts of Northern and Southern Germany (Fig. 5). The high values in France are due to particularly large numbers of CSAs and farmers’ markets (Fig. B3), driven by strong peasant and critical consumption movements that gave rise to the AMAP (association for maintaining small-scale family farming) (Urgenci 2016). Italian cultures also place strong emphasis on food and its quality (e.g. as the birthplace of the ‘slow food’ movement), so consumers are particularly interested in local products and short VCs (Fig. B3). As we measure civic food networks using only four indicators, the spatial results are more sensitive to the indicator selection (SM C) and using a min–max scaling leads to higher index values in several countries, due to very high relative prevalences of farm shops in Denmark and farmers’ markets in Portugal and Romania.

Civic food networks are weakest in Scandinavia, the UK, Portugal, and parts of Eastern Europe. The results for the UK and Scandinavia reflect the low levels of consumer willingness towards alternative food (Fig. B3), whereas Poland and Czechia have relatively few farm shops. This again highlights the challenge of identifying universally culturally appropriate indicators, as there is evidence of strong informal civic food networks in these places that is missed by the available datasets (e.g. urban food production and food sharing) (Bellows 2004; Jehlička and Daněk 2017).

The associations between civic food networks and contextual factors are similar to those for multifunctional VCs (i.e. wealthy, young farmers), but their enabling environments are also stronger in cropping systems (compared to livestock) (Table B1). As we assessed associations rather than causality, we can only speculate about the directionality of any relationships between the networks and these factors. For instance, a younger farmer population may be more willing to innovate and so may catalyse alternative agri-food networks. Alternatively, these networks may attract young farmers due to their ideology or lower financial barriers to entry.

Interestingly, civic food networks are associated with higher GHG emissions and excess nitrogen (Table B2). This may imply that civic food networks rise as a niche alternative in regions where the environmental costs of agriculture are highest. For example, France, which has the strongest enabling environments for civic food networks in our study, also has a history of high nutrient inputs (Demay et al. 2023) and regional agricultural landscape degradation (Gianoli et al. 2023). Such trends may motivate frustrated producers and consumers to organise to disrupt the incumbent regime (Rossi et al. 2019; Rutting et al. 2022).

### Overlapping lock-ins and enabling environments

**Fig. 6:**
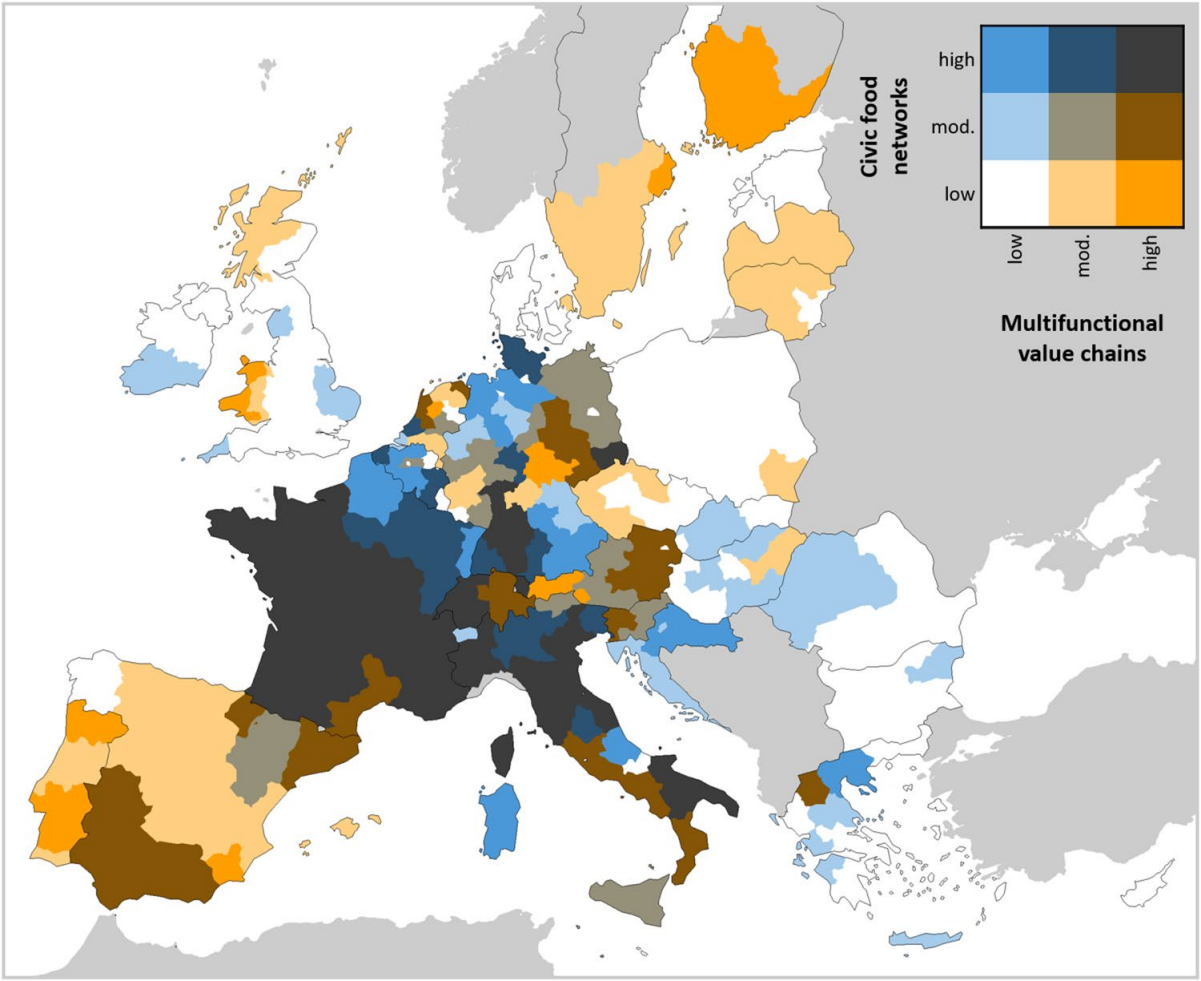
Alignment of enabling environments for *multifunctional value chains* and *civic food networks*. The legend groups each index by quantile into relatively low (0–50), relatively moderate (50–75), and relatively high (75–100). Note that this figure does not consider the strength of *agro-industrial control* (see Fig. 7)

The results highlight the bright spots of co-evolving alternative agri-food networks within Europe’s prevailing agro-industrial context (Fig. 6). While we cannot make absolute assessments, many regions in France, Italy, Switzerland, and SW Germany show relatively strong enabling environments for *both* alternative network types. Other regions have supportive environments for one network type only: multifunctional VCs (Spain, Portugal, Latvia, Finland, and Sweden) or civic food networks (Croatia, Belgium, and parts of Germany). However, many regions have weak enabling environments for both network types (Poland, Denmark, Luxembourg, Lithuania, Estonia, Bulgaria, Malta, UK, and Ireland).

These results provide a useful lens for considering regionalised pathways to sustainability (Bennett et al. 2021). The maps suggest that some regions are better positioned to follow market-based pathways (i.e. multifunctional VCs), whereas others may leverage a stronger role of civil society or grassroots movements (i.e. civic food networks). Because multifunctional VCs are a less radical alternative to the agro-industrial norm, it may be easier for them to scale (Duncan and Pascucci 2017), but there is a risk that their values are co-opted or diluted to undermine their overall transformative potential [e.g. organic agriculture (De Wit and Verhoog 2007)]. Civic food networks, in contrast, stand more radically in opposition to conventional VCs, and therefore have greater transformative potential but may struggle to see their practices adopted at larger scales (Duncan and Pascucci 2017). It may therefore in practice be beneficial to seek complementarities across region-specific capacities (shown in Fig. 6) by promoting hybrid network arrangements (Lamine et al. 2012). Transitions are complex and dynamic processes in which niche initiatives continuously redefine their objectives (Bui et al. 2016), navigate tensions between idealism and pragmatism (Poças Ribeiro et al. 2021), and enrol different kinds of actors (Vermunt et al. 2020). It remains an open question whether these network configurations will eventually generate systemic transitions or instead ‘fit and conform’ alongside the regime (De Schutter 2017; Smith and Raven 2012), but it is clear that regions will follow diverse pathways and our results provide an indication of the current directions of regional transitions in progress.

**Fig. 7:**
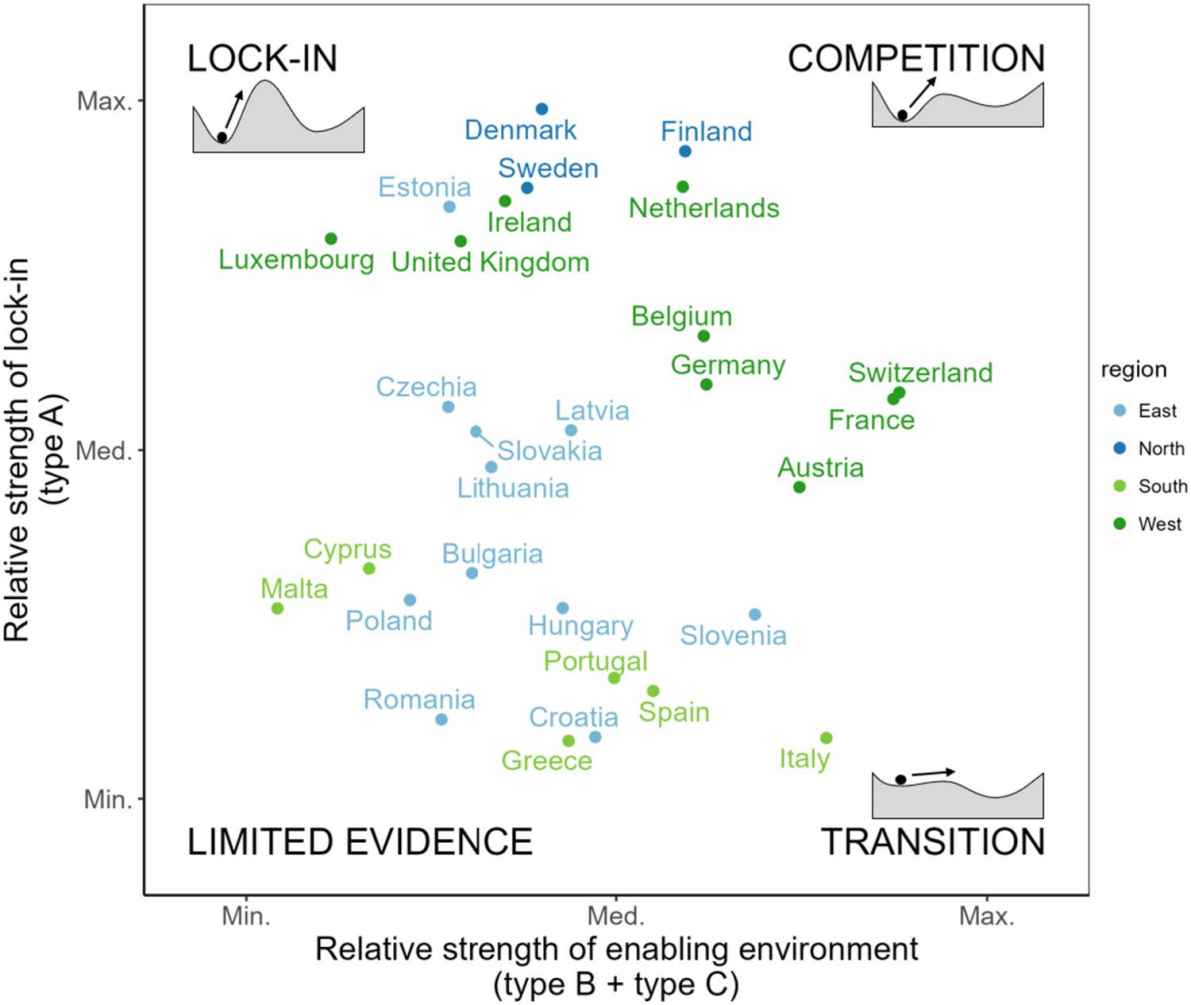
Relative transition potential for each European country. Here, the indexes for network types B and C are combined into a single enabling environment index (horizontal axis). Country-level values were calculated as the weighted average of their NUTS2-level indicators (weighted by € agricultural output)

When considered in conjunction with the relative strength of lock-in (Fig. 7), we can categorise regions (here: countries, for visual clarity) as demonstrating more or less potential for transitions towards sustainability. The countries most locked-in—Luxembourg, the UK, Estonia, Ireland, Sweden, and Denmark—currently have strong agro-industries and limited alternatives. These are therefore priority areas for public policy and investment (see Sect. “Targeting public policy for agri-food transitions”).

Italy is a frontrunner, with simultaneously low relative levels of lock-in and strong enabling environments for alternative agri-food networks. While this does not imply that alternative agri-food networks are *dominant* in Italy, we can infer that they have more strongly impacted regional agri-food systems. This is corroborated by the presence of ‘organic districts’ in Italy (Guareschi et al. 2023), which regionally embed components of both organic agriculture and agroecology. Italian municipalities also have a long history of sustainable forms of public food procurement (Sonnino 2009) and are global leaders in promoting sustainable urban food systems (Chrzan 2004).^1^

The Northern and Western European countries sit along a gradient between lock-in and competition, i.e. demonstrating strong agro-industries and variable strengths of alternative networks. In these places, niche innovations likely exhibit a degree of ‘hybridity’ (Lamine et al. 2012) by expressing both conventional and alternative characteristics. While it may be possible to successfully integrate these different paradigms (De Herde et al. 2020), there is an inevitable degree of competition (Sonnino and Marsden 2005) that must be considered when scaling innovations under conditions of strong agro-industrial control.

However, this analysis also raises an important question about the network typology: what does it mean to have low values for all indexes (i.e. “Limited evidence” in Fig. 7)? The countries in this position are mainly located in Southern and Eastern Europe, where less industrialised agricultural systems are still present. In these systems, external input intensity and engagement with large-scale VCs is relatively low, but there is also relatively little cooperation and market-based differentiation formally present in European-level datasets. It is possible that some farmers in these regions do have a high degree of collaboration and integration with civil society, but that these relationships are not captured by official datasets. This ultimately demonstrates that the network characterisation is not exhaustive and could be improved by including more traditional or subsistence-oriented network configurations (Marshall et al. 2021). It also suggests potential data limitations on alternative agri-food networks in these places. The degree of evidence for all three network configurations is associated with GDP (Table B1), suggesting that such data limitations are more present in lower-income countries.

### Targeting public policy for agri-food transitions

State actors have important roles to play in steering agri-food development, and already have a toolbox of instruments that can work to strengthen each network type (Table 4). Some correspond directly with our indicators. For instance, direct payments to farmers constitute the main component of the CAP Pillar I and align with agro-industrial control, as they are outside the choice of the farmer (i.e. indicate farmer passivity) and have historically facilitated input dependence (Linares Quero et al. 2022). This is where the majority of the EU’s funding is spent (Pe’er et al. 2020), suggesting that European agri-food policy has historically aligned most closely with agro-industrial control. Instruments that strengthen multifunctional VCs most frequently sit within the scope of the CAP Pillar II and focus on incentivising sustainable agriculture, promoting alternative VCs (e.g. with certification schemes or public food procurement), and facilitating more equitable power balances. It is indeed possible that farmers also become dependent on these instruments (e.g. payments for organic agriculture), but they are necessary in neoliberal global food systems that do not otherwise value non-economic qualities of food. Given the more prominent role of consumers in civic food networks, corresponding instruments focus less exclusively on farmers and agricultural production. The state has potentially fewer established mechanisms for governing these networks, suggesting the necessity to form multi-stakeholder coalitions (e.g. with consumers and CSOs) to strengthen civic food networks (Moberg et al. 2021; Rossi et al. 2019), while ensuring that marginalised actors have the agency to affect the coalitions’ agendas and decision-making (Montenegro de Wit et al. 2021).

**Table 4:** Policy instruments that reinforce each network type and may therefore be used to transform network structures over time (modified from Galli et al. (2020))

Network type	Instruments for strengthening this network type
A: Agro-industrial control	Subsidies and direct payments to farmers (CAP pillar I) Regulations and standards (e.g. for GMOs, hormones, pesticides, seed marketing) Market and trade regulation (e.g. import tariffs, taxation)
B: Multifunctional value chains	Rural development programmes (CAP pillar II; e.g. agri-environmental schemes, farm investment support, cooperation measures, quality products promotion, support for advisory services; LEADER) Legislation and support for alternative labels and certification schemes (e.g. marketing standards, organic, animal welfare labelling, PDOs/PGIs, traffic light labelling such as nutri score) Promotion campaigns, e.g. for local food products or organic food Public food procurement that supports short supply chains and sustainable production (e.g. fruit and milk schools’ scheme) Monitoring unfair trading practices and competition Fair labour rules Funding for research and innovation on agri-food sustainability (e.g. EIP-AGRI, Horizon Europe)
C: Civic food networks	Food education policies (e.g. school food policies, knowledge on healthy diets) Support to farmer–consumer networks, such as CSAs and participatory guarantee systems (e.g. Nature & Progrès in France) Support to agro-tourism and farm businesses Urban agriculture and short food supply chains Circular food economy Support for developing polycentric network infrastructure (e.g. regional seed production) and food policy councils

But how should state actors leverage these instruments to facilitate sustainability transitions? The spatial diversity observed in our results implies that context-sensitive governance is necessary. Such tailored public policy should follow two complementary approaches: (1) facilitate upscaling of alternative agri-food networks where they already exist and (2) strengthen the enabling environments where they do not exist. Both approaches imply redirecting funding away from supporting agro-industrial control.

Where enabling environments are already relatively strong, public policy should facilitate the expansion and institutional embedding of alternative agri-food networks. This corresponds with a shift from supporting efficiency gains and input substitution towards redesigning entire production–consumption systems (DeLonge et al. 2016). This can involve support for expanding VCs (Moschitz et al. 2021), as farmers often lack the necessary infrastructure to bring agricultural products to market (e.g. processing or storage facilities) (Gava et al. 2022). States can also play a role as VC actors themselves; public food procurement initiatives (e.g. in cafeterias for public employees, schools, universities, or hospitals) can integrate sustainability into their procurement criteria to facilitate regional agri-food sustainability (Stahlbrand 2016). Upscaling alternative agri-food networks also requires support for transferring knowledge and learning (Gava et al. 2022), both between European actors and from successful initiatives elsewhere [e.g. the Forever Green Partnership in the US Midwest (Jordan et al. 2023)]. The EU already has relevant programmes, such as EIP-AGRI and LEADER+, that provide financial and organisational support for connecting actors around innovation. These programmes should therefore be strengthened towards supporting the missions and values of alternative agri-food networks (Klerkx and Begemann 2020). Policy implementation can also be more effective by leveraging the strengths of existing multi-stakeholder initiatives, such as food policy networks (den Boer et al. 2023). Frontrunning regions could also promote deeper transformations of farming practices and power relations through arrangements such as CSAs or participatory guarantee systems (Cuéllar-Padilla and Ganuza-Fernandez 2018).

Where enabling environments are currently weak, public policy must contend with the extant power relations. Where weak enabling environments overlap with strong agro-industrial control, policies could aim to regulate VC corporations (e.g. through competition policy or labour regulations) (Clapp 2022; Österblom et al. 2022), while ensuring that regulatory and administrative burdens are not unduly passed onto farmers or others (Kuokkanen et al. 2017). As farmers have little autonomy in these networks, they may oppose additional regulations of their behaviour, as seen for example with livestock farmer protests to new nitrogen laws in the Netherlands (Holligan 2022). In conjunction to weakening lock-ins, public policy can create enabling environments for new networks to emerge. Policy instruments within this approach could include public advertising campaigns to increase citizen awareness about sustainable agriculture, funding for multi-stakeholder collaboration, or financial support to help farmers transition their management practices (Gava et al. 2022). These initiatives can together help to create spaces that empower actors to innovate and are protected from dominant forces and markets (Smith and Raven 2012).

The EU agri-food policy environment is currently changing, and these changes create opportunities for advancing the above approaches. The F2F strategy generally aims to reduce the dominance of agro-industrial control, for instance by reducing fertiliser and pesticide use and regulating business conduct. The proposals mostly relate to formalising sustainability considerations in markets and VCs, e.g. through carbon sequestration markets, expanding organic agriculture, and including sustainability and origin labelling on food packaging. The vision of the F2F therefore aligns most closely with multifunctional VCs and does not spotlight the potential roles of civil society or farmer–consumer networks in organising food systems. To be most effective, our study suggests that the implementation of the F2F should: (1) leverage context-specific regional capacities and therefore also (2) recognise the *multiple* pathways that regions may pursue (e.g. multifunctional VCs and civic food networks).

Of course, these recommendations prompt many further questions and challenges. (Sub)national governments may lack the capacity—or the will—to take action towards sustainability (Guyomard et al. 2023), and delegating authority away from the European level can lead to time-consuming negotiations that may dilute intended policy impacts (Moschitz et al. 2021). Further, the polycentric structure of civic food networks implies less top-down governance, raising questions about the appropriate role for state actors in such a transition. In a sense, civic networks emerge as conscious, counter-hegemonic protests against top-down control (Vivero-Pol 2017) and may actively resist state involvement. Reaching policy targets therefore requires a mix of binding, EU-wide instruments and flexibility to tailor solutions and re-distribute power to local contexts (Moschitz et al. 2021). Due to the multiple dimensions of sustainability, no single network configuration is inherently preferable and it may be best to leverage complementarities by promoting hybrid network types. Decisions about policy implementation will therefore inevitably involve value judgements, e.g. whether we should prioritise local food versus reducing GHG emissions. Our maps cannot help to make such value judgements, but by describing the diversity of European network contexts they could be used to guide conversations about multi-level governance.

### Data availability and methodological considerations

Our analysis synthesised the best available data, but this data is limited and therefore underscores a need for more comprehensive data collection on alternative agri-food networks. For instance, several indicators map the prevalence of relationships or infrastructure that we assume fosters cooperative behaviour (e.g. farmers’ membership in agricultural cooperatives, availability of farmers’ markets), but we cannot know if these initiatives effectively embody the values that they theoretically represent. The relevance of other indicators may be affected by attitude–behaviour gaps (Virginie et al. 2022), e.g. consumer attitudes do not necessarily translate to purchasing behaviour. The regional expenditure on state-led rural development schemes (e.g. EIP-AGRI, CAP Pillar II) may not represent their efficacy and misses additional regional or national programmes.

For these reasons, we consider the results to represent ‘enabling environments’, rather than a quantitative or absolute indication of each network type’s strength. The relative approach allowed us to avoid subjective judgements about what is ‘strong’ or ‘high’, but data on farmers’ marketing outlets or consumer purchasing behaviour (e.g. through supermarkets versus local shops or farmers’ markets) would enable assessments of the dominant agri-food network in each region. Further, explicit mapping of actors, such as specific companies and network-based initiatives, could provide more actionable entry points for decision-making.

The scope of this research is limited to agri-food systems and does not consider external trends (Debonne et al. 2022) or agriculture’s relationships with other land uses, such as forests or urban development. The relationships between these overlapping policy domains are central to many pressing concerns around biodiversity and climate change (Ortiz et al. 2021), and future research could expand our scope to consider networks of actors relevant to, for instance, forest or urban governance. The NUTS2 spatial unit is relevant for characterising regional networks and policy implementation, but misses both hyper-local dynamics and large-scale trade relations. Trade expands the spatial extent of an agri-food network (Kinnunen et al. 2020), but does not undermine the relevance of the regional focus, as the social and environmental impacts of agriculture mainly accrue in the location of production. Finally, our study did not attempt to infer causal relationships between agri-food networks and sustainability outcomes, so more research is needed to provide relevant evidence of causal mechanisms for environmental policy-making.

## Conclusions

Pathways to food system sustainability will differ between places and involve distinct actors and leverage points. Knowledge about the institutional environments that enable or hinder transition, and how they vary spatially, is therefore critical to designing effective, context-sensitive governance instruments. Our study provides the first spatial characterisation of alternative agri-food networks in Europe, and our results are relevant to both research and policy.

For researchers, our study has both practical and conceptual impacts. Practically, we provide the data that was synthesised for this study from a variety of creative sources (e.g. we created the first Europe-wide, subnational map of CSA prevalence). This data could be used to extend land-system classifications to encompass the institutional dimensions beyond land-use and land-management intensity (Kuemmerle et al. 2013), as well as to broaden farmer typologies to encompass network contexts (Bartkowski et al. 2022). Conceptually, agri-food networks may help to reconcile fragmented research domains by integrating individualistic perspectives on sustainability transitions [e.g. farmers’ decisions about technology adoption (Swart et al. 2023)] with notions of systemic (in)stability arising from actors’ interactions. For researchers studying farm management and individual behaviour change, our assessment characterises the broader network contexts that surround farmers and enable or restrict behaviour change. For researchers studying systemic lock-ins and transformations, our analysis demonstrates the spatial diversity and co-occurrences of *multiple* network paradigms, and could inspire investigations into the interactions between different networks, as well as the role of geography in mediating these interactions.

For governance of agri-food transitions, our results suggest that some European regions are better poised to follow market-based transition pathways (e.g. with formal labels like organic or PDO), whereas others have stronger civic involvement in food that could spur trade relations outside of formal VCs. Regions with both strong multifunctional value chains and civic food networks could foster increased collaboration between these network types to leverage their complementarities. Other regions, however, will require extra support and investment to strengthen their enabling environments for sustainability transitions through alternative agri-food networks. The maps and data show where these regions are located and could therefore inform context-sensitive agri-food policies across Europe.

### Supplementary Information

Below is the link to the electronic supplementary material.

Supplementary file1 (PDF 2025 KB)

## Data Availability

The data that support the findings of this study are openly available at DataVerse.nl under the following link 10.34894/P4QPIE.

## Footnotes

1 See also the Milan Urban Food Policy Pact https://www.milanurbanfoodpolicypact.org/.
