# Supplementary material for: Mapping lock-ins and enabling environments for agri-food sustainability transitions in Europe
Source: Sustain Sci. 2024 Apr 8;19(4):1221–42. doi: 10.1007/s11625-024-01480-y (PMC11245428; doi:10.1007/s11625-024-01480-y)
Supplement: Supplementary file 1 — Supplementary file1 (PDF 2025 KB) [file 11625_2024_1480_MOESM1_ESM.pdf]

Supplementary Material

Mapping lock-ins and enabling environments for agri-food sustainability transitions in Europe

Supplement A: Indicator calculation and data processing details

We utilised a large range of data sources to compile the indicators. Here, we introduce these data sources and describe the approaches for deriving the indicators from the raw data.

The selection of indicators was informed by recurrent processes observed in the empirical literature on European agri-food systems (Williams et al., 2023) as well as consultation with a set of regional academic experts. The ten experts who responded to the questionnaire stated expertise spanning the following countries: Austria, Belgium, Cyprus, Czechia, Denmark, France, Germany, Greece, Italy, Romania, Slovakia, Spain, and Sweden. The responses to the questionnaire resulted in the addition and deletion of several indicators and the identification of limitations associated with the indicators.

Excluded regions

Our analysis focuses on NUTS2 regions within the European continent (including the British Isles) that contain greater than 10% agricultural land. Several NUTS2 regions are thus excluded from our analysis, namely:

- City regions (AT13, BE10, CZ01, EL30, DE30, DE50, DE60, UKI3, UKI4, UKI5, UKI6, UKI7)
- Non-agricultural regions (FI1D, ITC3, SE31, SE32, SE33)

Geographic rescaling

Our analysis utilised the 2021 NUTS2 classification. Many input data layers utilised older NUTS classifications, and for these we utilised the change information documented at https://ec.europa.eu/eurostat/web/nuts/history. Three types of changes have occurred: renaming, splitting, and merging. Renamed regions were simply renamed. The values for split regions were distributed proportional to the area of each resulting region. The values for merged regions were aggregated, weighted by the area of each original region.

When the native spatial representation of the data did not correspond to the NUTS regions, we used the following procedures, which are essentially naive, area-based allocations. If the data represented an intensity (e.g., average input intensity), the target polygon's value was calculated as the spatially-weighted average of all overlapping source polygon values. If the data represented a sum (e.g., value of subsidies), we first calculated the fraction of overlap for each source polygon, and then set the target polygon's value as the sum of all source polygon values multiplied by these overlap fractions.

Farm Accountancy Data Network (FADN)

The FADN collects annual socioeconomic characteristics of a sample of farmers across Europe. Many variables are published publicly as "standard output" indicators on their website (https://agridata.ec.europa.eu/extensions/FADNPublicDatabase/FADNPublicDatabase.html). These are aggregated to the FADN regions (n=145), which generally align with the EU NUTS2 regions, but in some countries data is only reported at NUTS1 or NUTS0 (i.e., nationally). For all indicators derived from the FADN data, we averaged the standard output values across 2016-2020. The section "Geographic rescaling" describes how we aligned all data to a consistent spatial representation.

External input intensity [network type A]: This indicator incorporates specific costs for both crops (i.e., seeds and plants (both purchased and home-grown), fertilisers, crop protection products and other specific costs related to crop production) and livestock (i.e., feeding stuffs, veterinary costs and other livestock specific costs). The total economic value of such inputs is then normalised by the total value of crop and livestock output. Using the FADN Standard Output codes (FADN, 2020), the indicator corresponds to:

(SE284*SE025 + SE309*SE080) / (SE135 + SE206)

Indebtedness [network type A]: The total value of liabilities (i.e., the value at closing valuation of total of (long-, medium- or short-term) loans still to be repaid), relative to the farm net income:

SE485 / SE420

EUROSTAT

EUROSTAT publishes a wide variety of agricultural socioeconomic statistics at NUTS2 level on their website (https://ec.europa.eu/eurostat/data/database). We created the following variables using these data. In all cases, if data were not available for a particular region for the desired time period, we drew the value, where possible, from the next most recent time period. If this was not available, we drew from national-level EUROSTAT data. If this was not available, we created an analogue from FADN data.

Livestock (fraction of agricultural economy) [location factor]: Animal output (code 13000) as a fraction of the total output of the agricultural industry (code 18000). Data is from 2016 in the economic accounts for agriculture table ("agr_r_accts").

Total utilised agricultural area: UAA_HA from the farm indicators table ("ef_m_farmleg"). Data is from 2016. This value is used to calculate several other indicators.

Population density [location factor]: Population at 1 January in 2021 ("demo_r_d2jan" table; 2019 for the United Kingdom), divided by the area of the NUTS2 region.

GDP per capita [location factor]: GDP at current market prices ("nama_10r_2gdp") divided by the population used to calculate GDP ("nama_10r_3popgdp"). Average over 2018-2020. Data for the United Kingdom are from 2018.

Labour productivity [network type A]: Total regional output of the agricultural industry (code 18000 in table "agr_r_accts") divided by the total labour (annual working units) reported in "ef_lf_main" (as at 2016).

Low production diversity [network type A]: This indicator was calculated through three steps. First, using the data from the agricultural accounts table ("agr_r_accts"), we extracted the regional economic output for each product category at the finest scale of detail available (e.g., durum wheat, barley, sunflower, sugar beet, olives, wine, pigs). Second, because some product categories are inherently high-value (e.g., milk, wine), we then took the square root of all economic outputs in order to reduce these kinds of influences on the assessment. Third, we calculated the Herfindahl-Hirschman Index (HHI), a measure of concentration, across all product categories in each region. The HHI is typically used to represent corporate consolidation in a competitive marketplace (and indeed it forms a part of the VC concentration metric described below), but it also has relevance here for representing regional concentration of food production towards a few products. We also experimented with the Shannon diversity index, but this required assumptions about how to deal with "missing" values (e.g., if no wine is produced in Finland) as well as an inversion to translate it to a concentration metric. We opted for the HHI as it required fewer assumptions.

Organic farming [network type B]: Calculated as the fraction of farms that are organic (fully converted and under conversion) using 2020 data from "ef_lus_main". Data for the United Kingdom come from 2016.

OpenStreetMap (OSM)

We used the "osmdata" R package (Padgham et al., 2017) to query and download point-based data from OSM. After downloading the point data (on 26 July 2022), we then summed the number of features within each NUTS2 region. We used the following queries (which were partly inspired by the farmshop dataset at https://farmshops.eu/):

- "shop=farm" - a shop at a farm, selling farm produce: regional, seasonal, freshly harvested goods
- "amenity=vending_machine", filtering to those with the key "vending" equal to any of the following: animal_feed, bread, cheese, eggs, milk, sausages, potatoes, noodles, honey.
- "amenity=marketplace" - a public marketplace where goods and services are traded daily or weekly. These can represent farmers' markets.

However, OSM is known to contain geographic coverage bias (Thebault-Spieker et al., 2018). In our context, western Europe is more extensively mapped than eastern Europe. To mitigate these biases in the reporting of OSM data, we divided each of the above counts by the total number of mapped amenities (i.e., points with the key "amenity") in each NUTS2 region. The resulting indicators therefore

represent the relative abundance of each amenity in a region (e.g., the number of farmers' markets per mapped amenity). There is a risk that this leads to over-inflated values in regions with few mapped amenities, but it is the best approach given the available data.

Thus, the final indicators are calculated as:

- **Farm shops and vending machines [network type C]:** the combined number of farm shops and vending machines per mapped amenity in OSM.
- **Farmers' markets [network type C]:** the number of marketplaces per mapped amenity in OSM.

Common Agricultural Policy (CAP) payments

The CAP is divided into two main pillars that each have distinct policy focuses: Pillar I focuses on direct payments to farmers and market measures, whereas Pillar II supports rural development and environmental practices. While Pillar I contains many unconditional payments (e.g., based on farmed area), many of the measures in Pillar II are opt-in and non-compulsory, based on management practices. Within each pillar, payments are divided into more than 100 different measures, which are not necessarily 100% consistent between countries (Nicholas et al., 2021).

For this study, we take a simple approach and create an indicator for each CAP pillar. Pillar I aligns more closely with the agro-industrial control network type, as when unconditional subsidies comprise a substantial portion of the economy it can be inferred that farmers are dependent on these payments (Linares Quero et al., 2022), and farmer dependence is characteristic of type A. This choice was informed by narratives about European agri-food development, where for instance Finish farmers' incomes have been described as "heavily reliant on subsidies" (Kuokkanen et al., 2017, p. 937), with 40% of their income coming from subsidies in 2014 (including agri-environmental payments). Historical payments within CAP Pillar I that were based on output levels have also more broadly affected narratives within farming communities, pushing farmers "towards the quest for the highest achievable yield" (Vanloqueren & Baret, 2008, p. 442) Pillar II, conversely, aligns more closely with the multifunctional value chain network type, because (i) many instruments within this pillar promote multifunctional agriculture and landscapes and (ii) the payments are conditional so facilitate farmer innovation and thereby have a more enabling character.

We originally experimented with filtering to a subset of the most relevant payment measures, but this requires >100 subjective decisions (i.e., deciding about the relevance of each measure) and so we opted for the more simple and transparent approach. Nevertheless, it is impossible for us to discern the actual levels of farmer dependence or multifunctionality generated through these payments, so we remain cognisant of these indicators' limitations.

To calculate the indicators for **Subsidy intensity [network type A]** and **State-led rural development [network type B]**, we first respectively summed all Pillar I and Pillar II payments within each NUTS2 region using the dataset provided by Nicholas et al. (2021). This dataset represents the CAP payments at NUTS3-level as at 2015, with some data from 2014 and 2016. To convert these total economic payments

to *intensities*, we then divided these values by the economic size of the agricultural economy (using the code 18000 from the EUROSTAT table "agr_r_accts").

Some regions are missing in the Nicholas et al. (2021) dataset. We filled these gaps using comparable indicators constructed from the FADN standard outputs. For the Pillar I equivalent, we constructed an indicator representing the total direct payments (SE606: EU and national decoupled and coupled subsidies, except on rural development, costs and purchase of animals) divided by the farm total output (SE131: Total value of output of crops and crop products, livestock and livestock products and of other output, including that of other gainful activities of the farms). For the Pillar II equivalent, we used the values for support for rural development (SE624: Environmental subsidies+ LFA/ANC subsidies + other Rural Development payments including RD national payments), again divided by the farm total output.

EIP-AGRI operational groups

The EIP-AGRI website (https://ec.europa.eu/eip/agriculture/en/node.html) contains a database of active and past operational groups. Each entry is associated with one or more geographical locations (at the NUTS3-level). We downloaded the full set of operational groups (as at 10 August 2022), filtered out duplicate titles in each region (i.e., allowed the same title in multiple regions), and summed the unique number of projects per NUTS2 region (n=2416 total). We then calculated the **Agricultural innovations [network type B]** indicator by normalising these counts by the total number of farms reported in EUROSTAT (using the "ef_m_farmang" table).

EU Barometer

The EU Barometer surveys are conducted on a sample of the population and data are geolocated to the NUTS2 level. We utilised data from the 2022 survey titled "Europeans, Agriculture, and the CAP" (https://europa.eu/eurobarometer/surveys/detail/2665), and specifically the following questions:

- QA12 How important or not are the following factors in your decision to buy food products? (%)
    a. They come from a geographical area that you know?
    b. They respect local tradition and "know-how"
    c. They have a specific label ensuring the quality of the product
    d. They are part of a short supply chain, i.e. they are directly purchased from food producers or there are few intermediaries between food producers and consumers
- QA14.7 Do you agree or not with the following statements related to food products coming from « organic » agriculture…?
    a. They are difficult to find in the supermarkets, shops or markets in the area where you live (%)
- QA19.4 Please tell whether you agree or disagree with each of the following statements about agriculture and climate change?
    a. You are prepared to pay 10 more for agricultural products that are produced in a way that limits their carbon footprint (%)

For each question, we calculated the percentage of respondents within each region responding yes. For the **Consumer perceived organic access [network type B]** indicator, we inverted the responses to question 14.7 so that it represents the proportion of citizens who do not find it difficult to find organic food. To construct the **Consumer willingness towards alternative food [network type C]** indicator, we averaged the responses to questions 12a, 12b, 12d, and 19.4. The United Kingdom was not included in the 2022 survey, so we used data from the comparable 2018 survey.

Crop suitability

We calculated normalised values of arable **crop suitability [location factor]** (ranging between 0-1), based on the GAEZ database (FAO, 2023). In particular, we used the GAEZ suitability index maps under rainfed conditions. These maps combine relevant information on climate, soil, and topography to account for both agro-climatic potential yields and yield reduction factors due to the constraints induced by soil limitations and slope conditions. Using country-level information from Eurostat on the harvested area of arable crops (2013-2017; table “apro_cpshr”), we calculated a weighted average suitability value for each NUTS2 region. We only include the suitability of the following crops, which comprise at least 10% of the arable crop share in at least one country: wheat, maize, barley, oats, potatoes, rapeseed, and sunflower.

Community-supported Agriculture (CSA)

Urgenci (2016) conducted an extensive Europe-wide census of CSA schemes. In their report, they present the total number of CSAs and CSA consumers in each surveyed country (Figures EU2 and EU3 in their report). We collected further data to downscale their results to the NUTS2-level. Specifically, for the top ten countries (with more than 20 CSAs in 2015), we searched for national-level databases of CSA locations. For countries where this was available (all except Spain), we downloaded their spatialised CSA data and used this to provide a sub-national weighting of the Urgenci data (Table A1). For consistency across countries, we left all absolute numbers as reported by Urgenci, even if there were more CSAs reported on the country data portals.

Some NUTS2 regions are larger or more populated than others, so we normalised by dividing the number of CSA consumers in each NUTS2 region (from the Urgenci report and subnational re-weighting) by the population (using Eurostat data, described above). The final metric for **Community-supported agriculture [network type C]** represents the number of CSA consumers per million residents.

Some countries were not included in the Urgenci report (Portugal, Denmark, Luxemburg, Estonia, Latvia, Lithuania, Croatia, and Bulgaria). It is not explicitly described in the report why these countries were not included, but it is implied that Urgenci did not find evidence of CSA in these countries, or at least was unable to identify or contact any national organisation or spokesperson. We therefore interpret this exclusion as meaningful (i.e., not random) and assign values of zero to these countries. This of course likely underestimates the true presence of CSAs in these countries, but we choose to honour the reporting of Urgenci, as theirs is the most extensive evaluation of CSAs to-date.

Table A1. Sources of subnational CSA locations. All websites were accessed between 7-10 December 2022.

Country	Source(s)
United Kingdom	https://communitysupportedagriculture.org.uk/find-a-csa/
Czechia	https://kpzinfo.cz/
Italy	https://e-circles.org/mappa-gruppi-di-acquisto
Belgium	Integrated three sources:<br>http://csa-netwerk.be/word-deelnemer.asp<br>https://www.voedselteams.be/voedselteams<br>https://asblrcr.be/cartographie/
France	https://www.avenir-bio.fr/annuaire_amap.php
Austria	https://solawi.life/solawi-finden/
Germany	https://www.solidarische-landwirtschaft.org/solawis-finden/karte#/
Netherlands	https://csanetwerk.nl/kaart/
Switzerland	German-speaking:<br>https://www.regionalevertragslandwirtschaft.ch/verband/index.php/initiativen-sp-729906689<br>French-speaking: https://www.fracp.ch/les-paniers<br>Italian-speaking:<br>https://conpro.bio/wp-content/uploads/2022/10/produttori-2022.pdf

Value chain concentration

The VC concentration metric is an average of three values that each represent concentration in a different aspect of the VC: manufacture of food products, agricultural wholesale, and food retail. These data are all only available at national-level, but this is the relevant scale at which to measure such processes of consolidation.

Eurostat collates information about industrial activity through their Structural Business Statistics database. Using the data in tables "sbs_sc_sca_r2" and "sbs_sc_dt_r2", we stratified the economic activity into two groups representing firms with greater than or less than 50 employees (Eurostat's definition of "large" is greater than 50 employees). Then, we calculated the fraction of total economic activity that is conducted through large businesses. We did this for two industry classifications: "Manufacture of food products" (NACE r2: C10) and "Wholesale of agricultural raw materials and live animals" (NACE r2: G462). We retained the most recent value for each geographic region.

For retailer concentration, we directly used data from Van Dam et al. (2021), who calculated various measures of industry concentration in the European food industry. We utilised their estimates of the

Herfindahl-Hirschman Index (HHI) for the supermarket industry, as at 2017. Of the countries in our analysis, their dataset excludes Cyprus, Luxembourg, and Malta.

We then calculated a single index for **VC concentration [network type A]** (for each country) as the average quantile across these three metrics. We retained the countries that were missing data for a single metric.

We note that aggregating across different stages of the VC may overlook countries in which particular VC stages are concentrated. For example, Slovenia has a highly concentrated supermarket sector but low concentration in agricultural wholesale and food product manufacture. Yet the aggregation that we perform is mathematically equivalent to creating three separate indicators (each with ⅓ of the regular weight) and subsequently aggregating them. If these three indicators each received the full weight, differences in the assessment for network type A would indeed be observed, but we believe this would overinflate the VC influence in the construction of the combined index (i.e., VC concentration would comprise 3 out of 8 indicators). In any case, the implications are likely small as we observe strong correlations between the three component VC indicators (all Spearman correlations are >0.4, with p<1e-10).

Farmer cooperatives

Bijman et al. (2012) conducted an extensive analysis of farmer cooperatives in Europe. They calculated national- and sector-level estimates of cooperative market shares (i.e., the fraction of production in each country and sector that is marketed by cooperatives), presented in Table 4.1 of their report. The sectors include: dairy, pig meat, sheep meat, wine, olives, fruit & vegetables, sugar, and cereals. We exclude dairy from our analysis, as dairy cooperatives are frequently large-scale and/or transnational (Bijman et al., 2014), and do not always promote the kind of cooperative behaviour that we wish to measure. Cooperatives in other sectors may in some cases also align more closely with agro-industrial control, but membership is around 50% lower in all other sectors (36% on average, compared to 65% in dairy), suggesting that cooperative participation in non-dairy sectors is more likely to be an active decision of farmers, which is what network type B aims to measure. The final dataset of Bijman et al. (2012) excluded Romania, Bulgaria, and Luxemburg, and for these countries we estimated values (where possible) using data presented elsewhere in the same chapter of the report.

We downscaled their national metrics to the NUTS2-level. This served two purposes: a) to estimate the sub-national variability in cooperative participation from the available data and b) to exclude regions for which there is likely insufficient information. We followed the following procedure to calculate the **Farmer cooperatives [network type B]** indicator:

1. Using production data from Eurostat (from “agr_r_accts”), calculate the fraction of production in each NUTS2 region corresponding to each sector category in the cooperative dataset, i.e., match the Eurostat codes with the cooperative categories as follows: dairy (Eurostat code 12100), pig meat (11200), sheep meat (11400), wine (7000), olives (8000, 6500), fruit and vegetables (4000, 6100, 6200, 6300, 6400), sugar (2400), cereals (1000).

2. Multiply each NUTS2-level production fraction (from step 1) with the respective national-level cooperative fraction from Bijman et al. (2012). For example, if 20% of the regional production is sheep meat and 60% of national sheep production is through cooperatives, the result is 0.2*0.6 = 0.12 (i.e., 12% of the region's farmers are in a sheep cooperative).
3. Sum across all product categories to estimate the overall cooperative prevalence in each NUTS2 region.
4. Exclude regions that have less than 20% of their total production value represented.

As some product categories are missed in the Bijman et al. (2012) dataset, this produces a low-range estimate of the prevalence of agricultural cooperatives in each region. The cooperative dataset also excludes Switzerland and Croatia. Unfortunately, we were unable to fill the Swiss data gap, even after personal communication with a Swiss expert on cooperatives. For Croatia, we utilised the outputs of an independent analysis of 2015 data (Pejnović et al., 2017) as follows. First, we calculated the total number of cooperatives in each NUTS2 region by aggregating the republic-level data presented in (Pejnović et al., 2017, p. 43) (HR02=232, HR03=220, HR05=20, HR06=59). Then, we calculated the number of cooperative *members* in each NUTS2 region by multiplying these values by the average number of members per cooperative (Pejnović et al. (2017) report 531 total cooperatives with a total of 8061 members and 170 member-employees, so 15.5 members per cooperative on average). Finally, we divided this by the total number of farms in each region to yield the fraction of farms that are members of agricultural cooperatives.

We also attempted to utilise data from personal communication with a representative of COPA-COGECA (the umbrella organisation for agricultural cooperatives in Europe), but their data did not cover all European countries.

Protected designation of origin (PDO)

Flinzberger et al. (2022) synthesise data to present maps of the number of registered PDO products in each NUTS3 region. Their analysis focuses on food products and therefore excludes wine. From their outputs, we calculate the number of unique PDOs in each NUTS2 region.

Socio-environmental megatrends and risks

Debonne et al. (2022) analysed the megatrends facing European agriculture that may affect the resilience of current agricultural systems. We examine how our network types associate with some of these megatrends. In particular, we construct a **climate change risk** indicator using their data, which represents regions that face either decreasing potential yields or increasing drought risk under climate change (this is therefore a binary metric).

We also use their concept of the environmental action space to examine the level of **excess nitrogen** as an environmental policy gap, representing soil surface surplus nitrogen, based on the results of an

integrated assessment model (5 arcmin raster, averaged over each NUTS2). We directly utilised the data from Debonne et al. (2022).

We constructed a further environmental metric to replace the GHG reduction pressure metric in Debonne et al. (2022), which was only available at national-level. Using the EDGAR dataset (https://edgar.jrc.ec.europa.eu/dataset_ghg60), we derived a metric that represents the **agricultural GHG emissions** in tons of CO2-equivalents per hectare per year. There are ten sectors in the EDGAR database that relate to agriculture:

- Enteric fermentation (CH4)
- Manure management (CH4, N20)
- Agricultural waste burning (CH4, CO2, N20)
- Agricultural soils (CH4, CO2, N20)
- Indirect N20 from agriculture (N20)

We summed the emissions (in CO2-equivalents) across these ten sectors for each pixel in the EDGAR database. Then, as the EDGAR database does not distinguish agricultural land from other land, we used the following process to convert these data to the NUTS2-level:

1. Calculate the fraction of each NUTS region that is used for agriculture (i.e., the utilised arable area as a fraction of the total region's area) (using Eurostat data)
2. Sum the emissions across the respective fraction of pixels with the highest agricultural emissions (e.g., if the UAA fraction is 0.4, select the top 40% of pixels within that region).
3. Divide the total resulting emissions by the UAA

Perpiña Castillo et al. (2021) examine future **land abandonment risk** across Europe using a utility-based modelling framework. In their study, land abandonment is a process of disinvestment in agricultural land due to changing economic benefits. Using their outputs, we calculate the fraction of land in each NUTS2 region that is at risk (moderate, high, or very high) of land abandonment.

We use the data compiled by the World Resources Institute on water risk (Hofste et al., 2019) to generate a **water stress** metric. We utilise their measure of baseline water stress, which represents "the ratio of total water withdrawals to available renewable surface and groundwater supplies" (source: https://www.wri.org/applications/aqueduct/water-risk-atlas/). This measure includes non-agricultural water uses (e.g., domestic and industrial), but is still relevant for our purposes because a) agriculture is a dominant water user in many regions and b) even if agriculture is not a dominant user, regional water stress sets the action space in which agricultural water use can operate. Their data classifies regions into discrete levels of water stress (low, low-medium, medium-high, high, extremely high). We attribute numeric values to each category and calculate the average water stress level per NUTS2 region.

Filling Swiss data gaps

As Switzerland is not in the European Union, many of the datasets described above do not cover Switzerland. We filled these gaps, to the best of our ability, using the following procedures and data sources.

We downloaded data from the regional agricultural accounts of the Bundesamt für Statistik for 2016-2020 (table px-x-0704000000_131).
https://www.pxweb.bfs.admin.ch/pxweb/de/px-x-0704000000_131/px-x-0704000000_131/px-x-0704000000_131.px/

- The **input intensity** indicator was calculated as the sum of costs for seeds and seedlings (C1.2.01), fertilisers and soil improvers (C1.2.03), plant treatment and pest control products (C1.2.04), vet and medicines (C1.2.05), and feed (C1.2.06), divided by the total production of agricultural goods (C1.1.01.1).
- Net income was calculated using the variable for net corporate profit (C3.5). Swiss Francs were converted to Euros, where applicable, using 2016-2020 exchange rates.

For **organic farming**, we calculated the percent of farms in each region that are organic, using 2016-2019 data from the Swiss statistical office (table px-x-0702000000_101).
https://www.pxweb.bfs.admin.ch/pxweb/en/px-x-0702000000_101/px-x-0702000000_101/px-x-0702000000_101.px).

To calculate the equivalents of the CAP Pillar I and Pillar II payments, we used data from the BLW at https://www.agrarbericht.ch/de/politik/direktzahlungen/finanzielle-mittel-fuer-direktzahlungen. The **subsidy intensity** indicator was calculated as the "security of supply direct payments" (Versorgungsicherheitsbeiträge), divided by the total agricultural output, which was drawn from the regional agricultural accounts (variable C1.1.01). The **state-led rural development** indicator was calculated as the sum of several other payments (cultural landscapes, biodiversity, landscape quality, production system, and resource efficiency), also divided by the total agricultural output.

The **consumer willingness towards alternative food** indicator was calculated by integrating estimates from several sources. We averaged the four values described below (87%, 65%, 79%, 80%) to yield a national-level estimate for the consumer willingness indicator.

- A survey conducted by the Swiss Retail Foundation in 2019 (Swiss Retail Foundation, 2019) found that sustainability is not important for only 13% of Swiss consumers, implying that it is important for 87% of consumers.
- In surveys conducted by the FiBL (Forschungsinstitut für biologischen Landbau), 65% of respondents said that regional origin is an important factor (Stolz et al., 2022).
- In an industry report utilising survey data from a 2021 survey (Deloitte, 2021), 79% of respondents said that sustainability concerns have some effect on their purchasing decisions. Further, 80% of people were prepared to pay 10% extra (or more) for sustainable food.

The **consumer perceived organic access** indicator was determined using expert judgement. Switzerland has one of the largest (by fraction) organic retail markets in Europe (FiBL, 2020), so we assume that it is high throughout. Specifically, we assume a value between Denmark and Sweden, as Switzerland's organic market share lies between these two countries in the FIBL-AMI 2020 survey. Therefore, we calculate Switzerland's (national-level) consumer perceived organic access indicator by averaging the respective indicators across all Danish and Swedish NUTS2 regions.

The **indebtedness** indicator was calculated based on the "Income Situation sample" of the Swiss FADN (Renner et al., 2019), which is representative of the target population of Swiss commercial farms. It was calculated as the total liabilities divided by the farm net income.

The **protected designation of origin (PDO)** indicator was calculated by counting the number of labels per canton and converting this to NUTS2 regions, using the data at https://www.aop-igp.ch/en/products.

We were unable to create Swiss equivalents for the agricultural cooperatives and EIP-AGRI scheme indicators.

Supplement B: Additional results

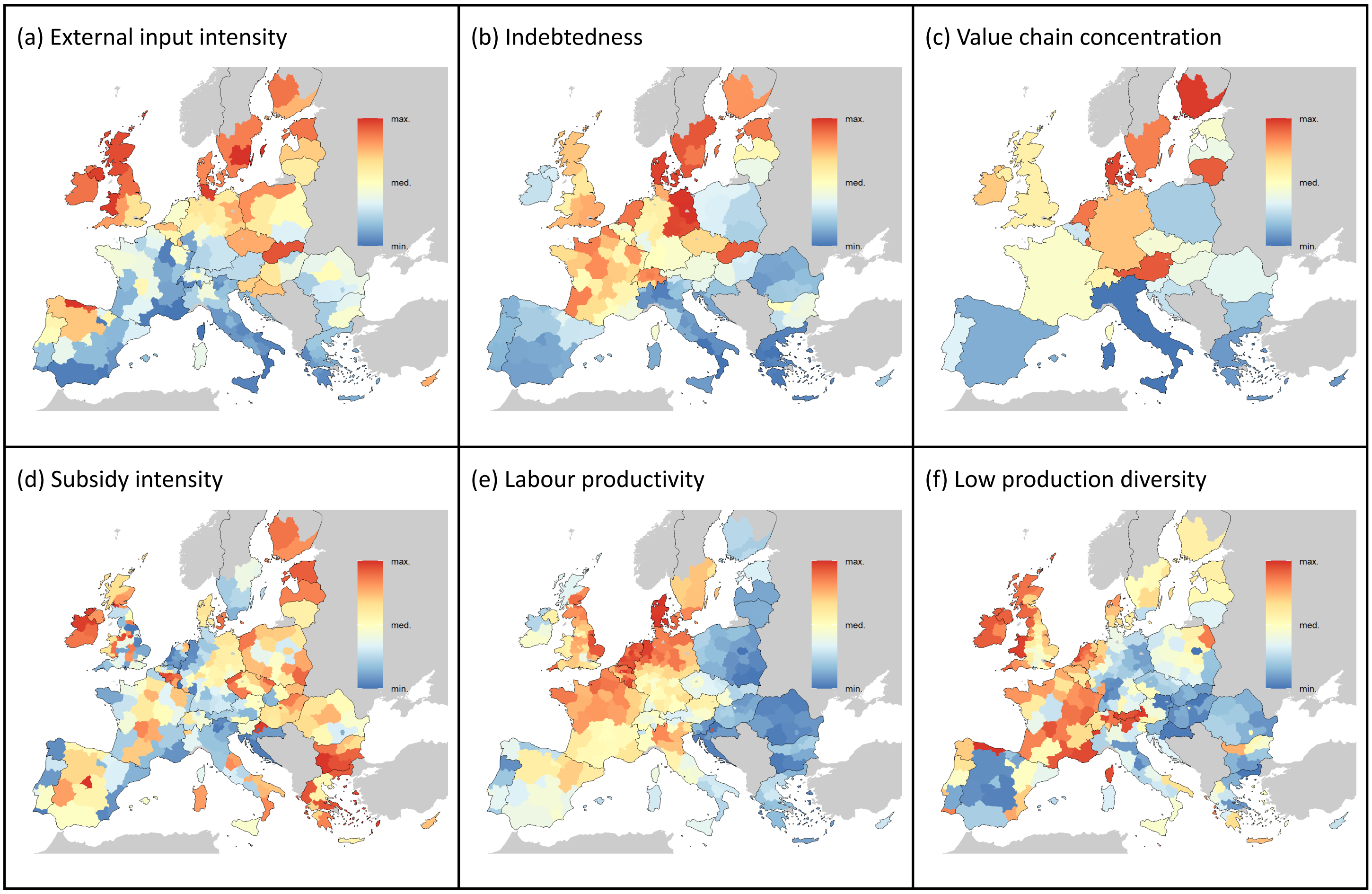

Figure B1. Individual indicator maps for *agro-industrial control*.

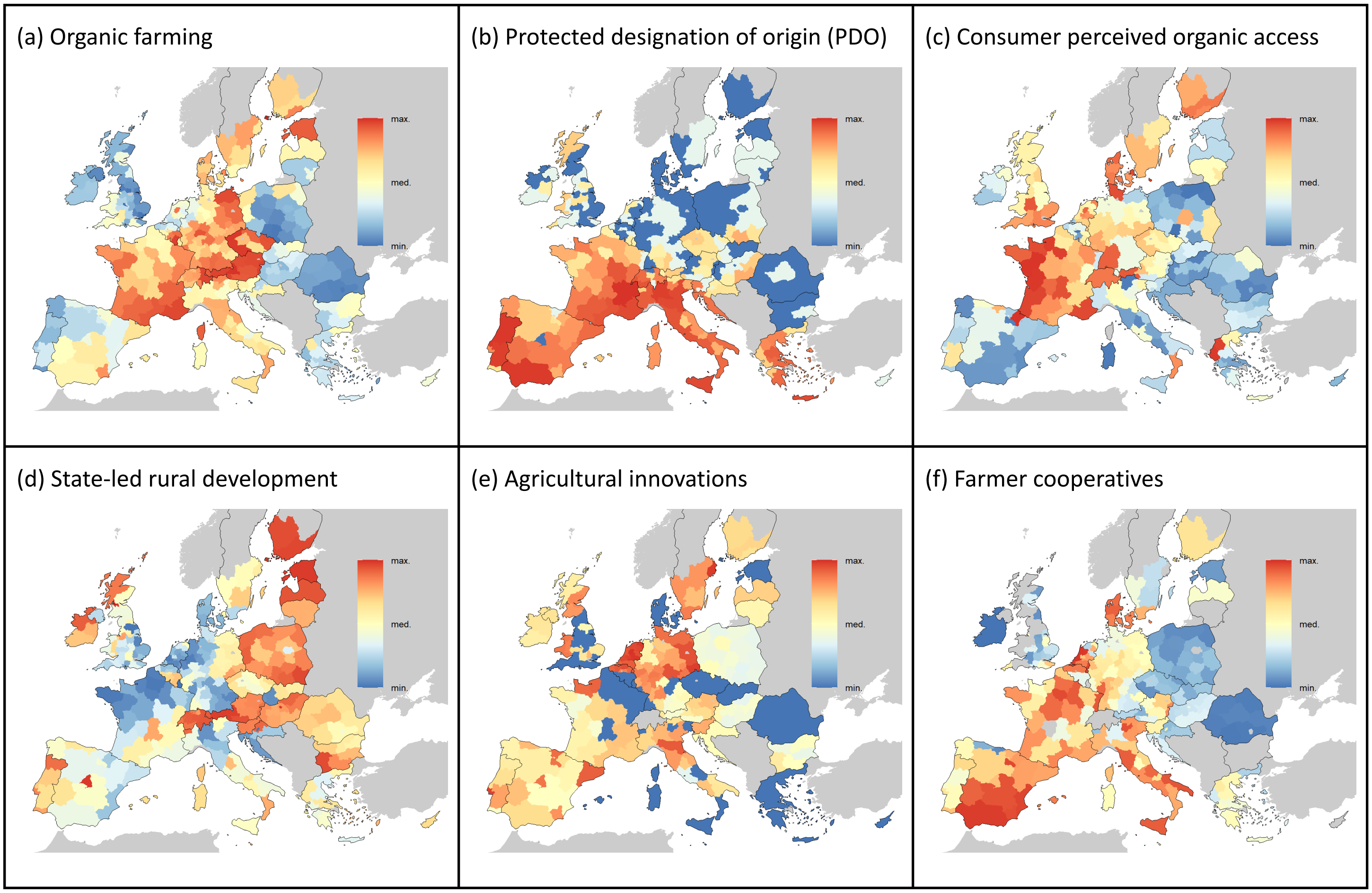

Figure B2. Individual indicator maps for *multifunctional value chains*.

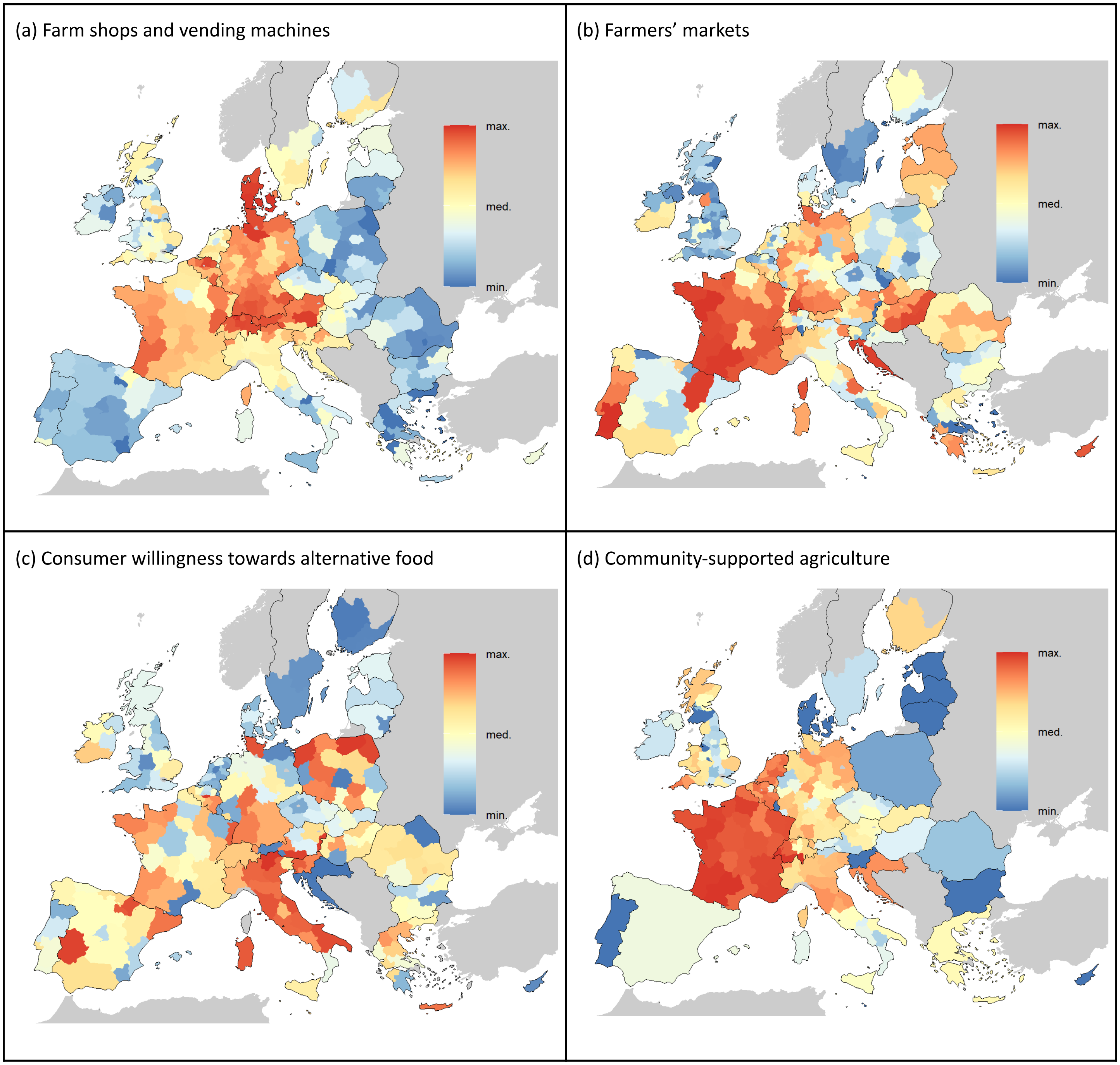

Figure B3. Individual indicator maps for *civic food networks*.

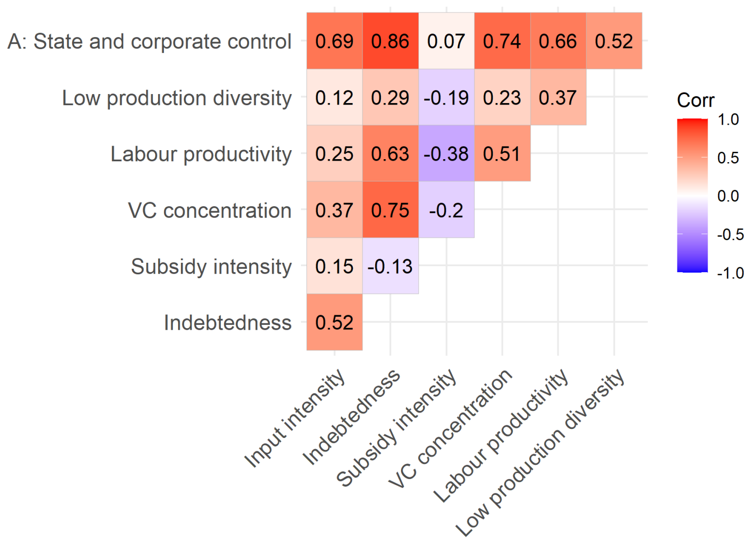

Figure B4. Spearman correlations between the indicators that make up network type A.

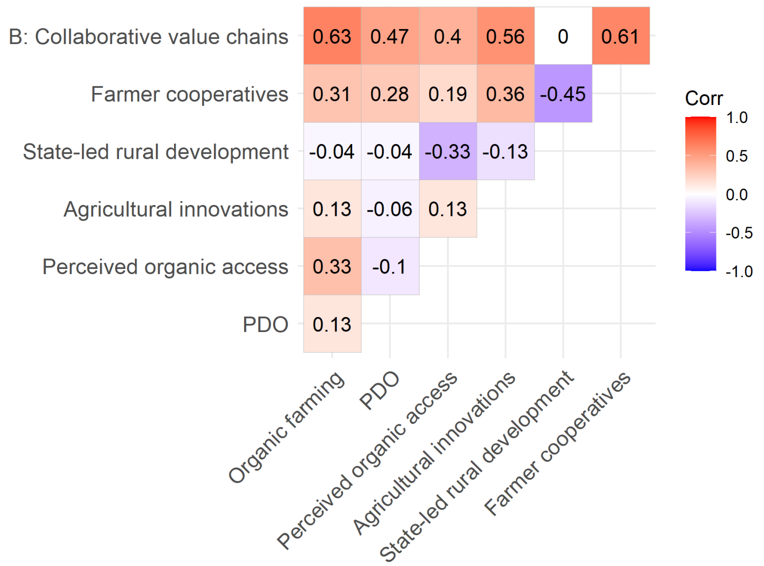

Figure B5. Spearman correlations between the indicators that make up network type B.

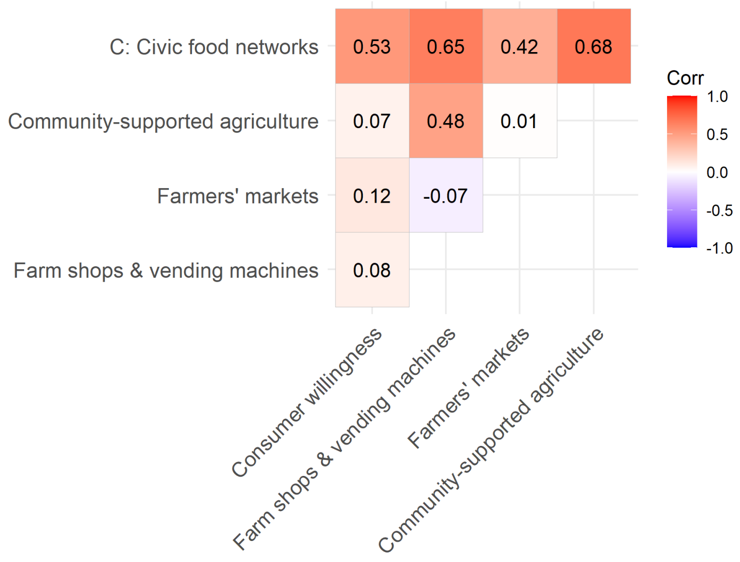

Figure B6. Spearman correlations between the indicators that make up network type C.

Table B1. Spearman correlations between network indexes and location factors. Only correlations significant at a 10% level are shown. (.) <0.1, * < 0.05, ** < 0.01, *** < 0.001.

	Crop suitability	GDP per capita	Population density	Livestock (frac. of economy)	Age ratio
**A: Agro-industrial control**	-	0.46 ***	0.24 ***	0.48 ***	-0.24 ***
**B: Multifunctional value chains**	-0.23 ***	0.42 ***	-	-	-0.27 ***
**C: Civic food networks**	0.13 *	0.29 ***	-	-0.15 *	-0.29 ***

Table B2. Spearman correlations between network indexes and socio-environmental megatrends. Only correlations significant at a 10% level are shown. (.) <0.1, * < 0.05, ** < 0.01, *** < 0.001.

	Environmental policy gaps		Other risks		
	**GHG emissions**	**Excess nitrogen**	**Climate risk**	**Land abandonment risk**	**Water stress**
**A: Agro-industrial control**	0.47 ***	0.64 ***	-0.16 *	-0.39 ***	-0.35 ***
**B: Multifunctional value chains**	-	-0.1 (.)	-	0.11 (.)	-
**C: Civic food networks**	0.23 **	0.15 *	-	-	0.16 *

Supplement C: Sensitivity analysis and validation

C.1 Sensitivity analysis

The first sensitivity analysis sought to examine the sensitivity of the spatial results to the indicator selection. The analysis proceeded as follows:

1. For each indicator (*i*) within each network type (*netw*)
   a. Exclude indicator *i* and recalculate the network index ($I_{excl,i}$)
   b. Double the weight of indicator *i* and recalculate the network index ($I_{double,i}$)
2. Calculate the sensitivity as the mean absolute difference resulting from this procedure:

$$Sensitivity_{netw} = 1/(2N) \sum_{i=1}^{N} (|I - I_{excl,\, i}| + |I - I_{double\; i}|)$$

Figure C1 shows the resulting sensitivity maps for each network type. In general, the differences are relatively minor, with average mean absolute differences of 5%, 9%, and 9% for each network type respectively. As there are only four indicators within network type C, each indicator has a stronger influence on the final result and therefore the sensitivity is highest (and particularly in Eastern and Southern Europe, where the indicators are highly variable (i.e., they score high on some type C indicators but low on others)). The hotspot in Switzerland for network type B is because Switzerland is missing data for two type B indicators, so their modification (exclusion/doubling) has a relatively large effect on the resulting network index.

(a) Agro-industrial control	(b) Multifunctional value chains	(c) Civic food networks

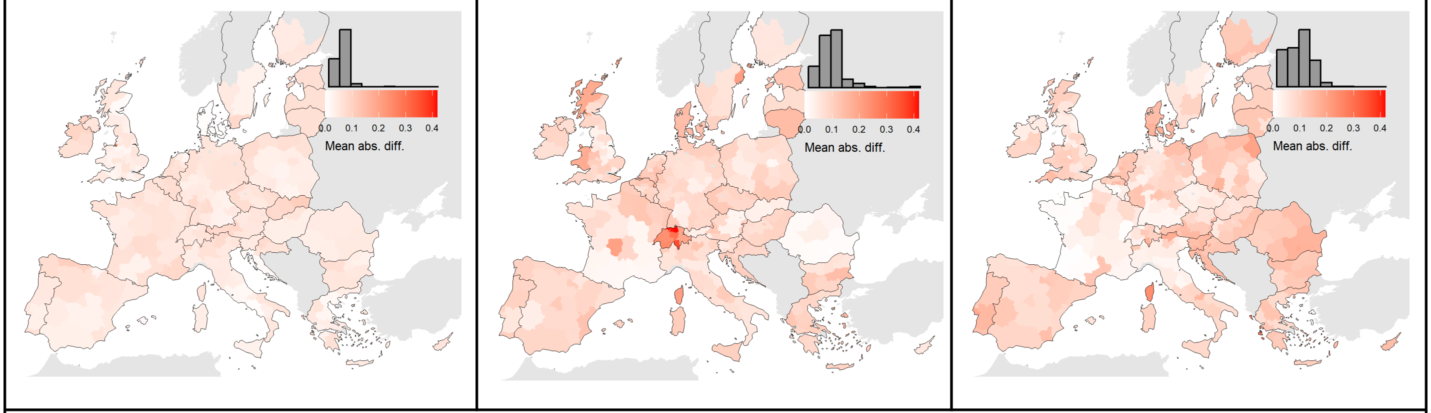

Figure C1. Sensitivity to indicator selection. The maps show the absolute difference in index value when each constituent indicator is both removed and doubled in weight, averaged over all constituent indicators.

The second sensitivity analysis examined the effect of the normalisation procedure. In the main analysis, we transformed all indicators to the same range (between 0 and 1) by taking the quantile. The quantile approach preserves the rank of the indicator values, i.e., going from 5th to 10th highest is equivalent to going from 155th to 160th. This is relevant given our interest in identifying the regions that are relatively high or low in Europe (e.g., the top 25% of regions), even when there are outliers for an indicator (e.g., if

the top value is twice as large as the second-highest). An alternative approach is min/max scaling, which scales all values between the minimum (0) and maximum (1), while preserving the scale of the original values, e.g., a change from 0-5kg/ha is equivalent to a change from 100-105kg/ha.

In this sensitivity analysis, we re-ran the index calculation with a min/max scaling and examined how the indexes changed (Figure C2). Again, network type C had the highest sensitivity overall (the mean absolute change in indicator value is 15%). This is because three of the four indicators within type C have long-tailed distributions (i.e., outliers at the top end), so the min/max scaling approach increases the index values for these regions compared to the quantile approach. These outliers include large numbers of farmers' markets in southern Romania and Portugal and lots of farm shops in Denmark. Croatia is an outlier with very low levels of consumer willingness towards alternative food, so the min/max scaling substantially decreases its overall index value. The sensitivity for networks A and B is relatively low.

(a) Agro-industrial control	(b) Multifunctional value chains	(c) Civic food networks

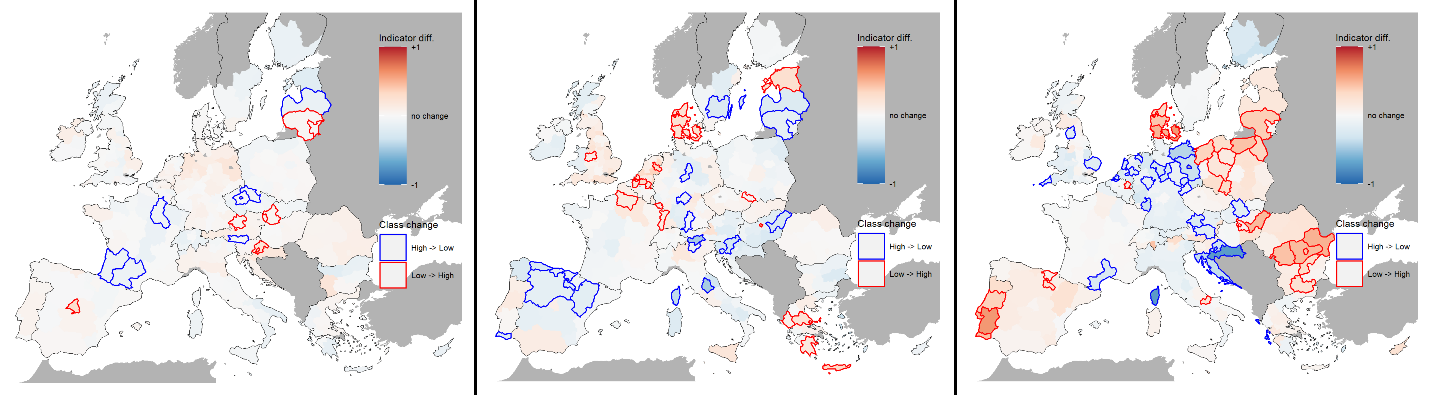

Figure C2. Sensitivity to standardisation method. The color gradient (i.e., polygon fill) represents the difference in index value when the min-max scaling approach is used, relative to the default quantile method. The polygon outlines represent the regions that change in classification from high to low (blue) and low to high (red) when the min-max scaling approach is used. High and low are respectively defined as above and below the median.

C.2 Validation with empirical case studies

A recent study characterised the agri-food networks described in qualitative case study research across Europe (Williams et al., 2023). Their study comprised 71 case studies at different scales, 64 of which could be spatially located to either a national level (n=26) or NUTS2 level (n=38) (Figure C3). Comparing their network classifications against the spatial indexes from this study serves as a partial validation of the mapping exercise; if the mapped indexes align with the case study classifications, this is evidence that the spatial indicators are together able to identify regions empirically classified as exhibiting that network type. The validation is only partial, however, as the case study research itself is not representative: neither does it systematically cover all locations, nor does a case study in a particular location necessarily represent that region as a whole.

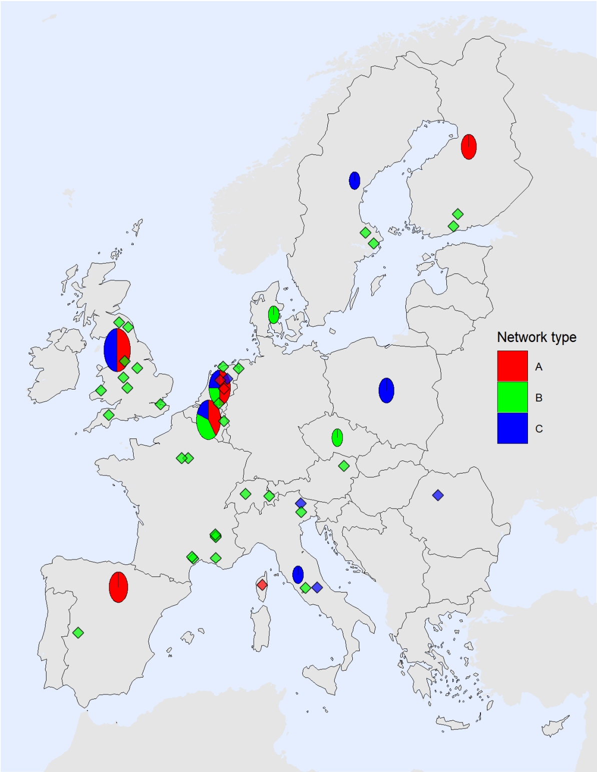

Figure C3. Locations and classifications of empirical case studies. Local-level case studies are plotted as points (diamonds). National-level case studies are shown as pie charts, with the size corresponding to the number of studies.

Nevertheless, our analysis aligns favourably with the empirical case study classifications (Figure C4), particularly at the local/regional level. These case studies most frequently characterised multifunctional value chains (type B; n=31), and these NUTS2 regions indeed had high indexes for type B in the mapping exercise (the 66th quantile on average). Local/regional case studies less frequently described type A and C networks (n=3 and n=4, respectively), but these locations also had relatively high mapped index values (72nd and 70th quantiles, respectively).

For the national-level case studies, the mapped indexes do not always align so well with the empirical classifications. The agreement for type A is good: countries in which empirical case studies describe A-type networks also have high mapped indexes for type A (77th percentile, on average). However, countries with empirical descriptions of type B and C networks contain *low* mapped indexes for these network types (i.e., below the median in Figure C4). These discrepancies, however, are easy to explain; these case studies include, for example, descriptions of civic food networks like farmers' markets and permaculture communities in the United Kingdom (Kirwan, 2004; Maye, 2018) (Figure C3), a country with low levels of civic food networks overall. Our interpretation of these discrepancies is therefore not that our mapping is inaccurate, but that research of type B and C networks has a tendency to focus on a biased set of locations – "bright spots" within otherwise industrialised agri-food systems – due to their academic interest as outliers from which to learn about agri-food transitions.

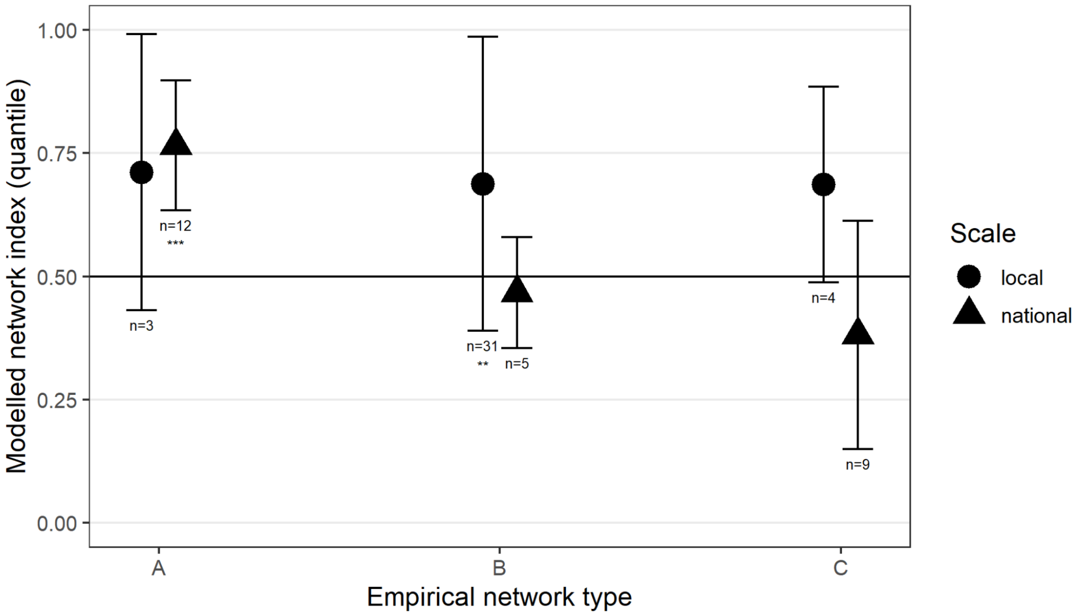

Figure C4. Alignment of the mapped indexes and classified empirical case studies. Points represent the mean mapped index corresponding to the empirical network type (e.g., index for type B when the empirical network is classified as type B), error bars show the standard deviation across the sample. Stars indicate the statistical significance of a two-sided Wilcoxon rank sum test that examines the difference of the median from 0.5 (***<0.001, **<0.01, all others >0.1). In this figure, points above the median (y>0.5) indicate good alignment.

References

Bijman, J., Iliopoulos, C., Poppe, K' Gijselinckx, C, Hagedorn, K., Hanisch, M., Hendrikse, G. W. J., Kühl, R., Ollila, P., Pyykkönen, P., & van der Sangen, G. (2012). *Support for Farmers' Cooperatives: Final Report*. library.wur.nl. https://library.wur.nl/WebQuery/wurpubs/fulltext/245008

Bijman, J., Pyykkonen, P., & Ollila, P. (2014). Transnationalization of agricultural cooperatives in Europe. *Dovenschmidt Quarterly*, 168.

Debonne, N., Bürgi, M., Diogo, V., Helfenstein, J., Herzog, F., Levers, C., Mohr, F., Swart, R., & Verburg, P. (2022). The geography of megatrends affecting European agriculture. *Global Environmental Change: Human and Policy Dimensions*, *75*, 102551.

Deloitte. (2021). *Sustainable food What Swiss consumers expect from companies and policymakers*. https://www2.deloitte.com/ch/en/pages/consumer-business/articles/sustainable-food.html

FADN. (2020). *Definitions of Variables used in FADN standard results* (Version RI/CC 1750). European Commission.

FAO. (2023). *FAO Global Agro Ecological Zones* [dataset]. http://www.fao.org/gaez/

FiBL. (2020). *European organic market grew to 40.7 billion euros in 2018*. FiBL. https://www.fibl.org/en/info-centre/news/european-organic-market-grew-to-40-7-billion-euros-in-2018

Flinzberger, L., Zinngrebe, Y., Bugalho, M. N., & Plieninger, T. (2022). EU-wide mapping of "Protected Designations of Origin" food products (PDOs) reveals correlations with social-ecological landscape values. *Agronomy for Sustainable Development*, *42*(3), 43.

Hofste, R., Kuzma, S., Walker, S., Sutanudjaja, E., Bierkens, M., Kuijper, M., Faneca Sanchez, M., Van Beek, R., Wada, Y., Galvis Rodríguez, S., & Reig, P. (2019). Aqueduct 3.0: Updated decision-relevant global water risk indicators. *World Resources Institute*. https://doi.org/10.46830/writn.18.00146

Kirwan, J. (2004). Alternative strategies in the UK Agro-food system: Interrogating the alterity of farmers' markets. *Sociologia Ruralis*, *44*(4), 395–415.

Kuokkanen, A., Mikkilä, M., Kuisma, M., Kahiluoto, H., & Linnanen, L. (2017). The need for policy to address the food system lock-in: A case study of the Finnish context. *Journal of Cleaner Production*, *140*, 933–944.

Linares Quero, A., Iragui Yoldi, U., Gava, O., Schwarz, G., Povellato, A., & Astrain, C. (2022). Assessment of the Common Agricultural Policy 2014–2020 in Supporting Agroecological Transitions: A Comparative Study of 15 Cases across Europe. *Sustainability: Science Practice and Policy*, *14*(15), 9261.

Maye, D. (2018). Examining Innovation for Sustainability from the Bottom Up: An Analysis of the Permaculture Community in England. *Sociologia Ruralis*, *58*(2), 331–350.

Nicholas, K. A., Villemoes, F., Lehsten, E. A., Brady, M. V., & Scown, M. W. (2021). A harmonized and spatially explicit dataset from 16 million payments from the European Union's Common Agricultural Policy for 2015. *Patterns (New York, N.Y.)*, *2*(4), 100236.

Padgham, M., Rudis, B., Lovelace, R., & Salmon, M. (2017). osmdata. In *The Journal of Open Source Software* (Vol. 2, Issue 14). The Open Journal. https://doi.org/10.21105/joss.00305

Pejnović, D., Radeljak Kaufmann, P., & Lukić, A. (2017). Development and contemporary characteristics of agricultural cooperatives in the area of Croatia. *Croatian Geographical Bulletin*, *78*(2), 5–48.

Perpiña Castillo, C., Jacobs-Crisioni, C., Diogo, V., & Lavalle, C. (2021). Modelling agricultural land abandonment in a fine spatial resolution multi-level land-use model: An application for the EU. *Environmental Modelling & Software*, *136*, 104946.

Renner, S., Jan, P., Hoop, D., Schmid, D., Dux, D., Weber, A., & Lips, M. (2019). Survey system of the Swiss Farm Accountancy Data Network with two samples: income situation sample and farm management sample. *Agroscope Science*, *68*, 1–76.

Stolz, H., Meier, C., Richter, S., Steiner, V., & Lupatsch, M. (2022). *Biobarometer Schweiz 2020 –Teil 1*. FiBL. https://orgprints.org/id/eprint/43844/1/Biobarometer2020-Ausfuehrliche_Foliensammlung_Ergebnisse.pdf

Swiss Retail Foundation. (2019). *LADEN LIEGT NOCH VORN – WIE LANGE NOCH?* Swiss Retail Foundation. https://www.swiss-retail.ch/news/laden-liegt-noch-vorn-wie-lange-noch/

Thebault-Spieker, J., Hecht, B., & Terveen, L. (2018, January 7). Geographic Biases are "Born, not Made." *Proceedings of the 2018 ACM Conference on Supporting Groupwork*. GROUP '18: 2018 ACM Conference on Supporting Groupwork, Sanibel Island Florida USA. https://doi.org/10.1145/3148330.3148350

Urgenci. (2016). *Overview of Community Supported Agriculture in Europe*. https://urgenci.net/wp-content/uploads/2016/05/Overview-of-Community-Supported-Agriculture-in-Europe-F.pdf

Van Dam, I., Wood, B., Sacks, G., Allais, O., & Vandevijvere, S. (2021). A detailed mapping of the food industry in the European single market: similarities and differences in market structure across countries and sectors. *The International Journal of Behavioral Nutrition and Physical Activity*, *18*(1), 54.

Vanloqueren, G., & Baret, P. V. (2008). Why are ecological, low-input, multi-resistant wheat cultivars slow to develop commercially? A Belgian agricultural "lock-in" case study. *Ecological Economics: The Journal of the International Society for Ecological Economics*, *66*(2-3), 436–446.

Williams, T. G., Bui, S., Conti, C., Debonne, N., Levers, C., Swart, R., & Verburg, P. H. (2023). Synthesising the diversity of European agri-food networks: A meta-study of actors and power-laden interactions. *Global Environmental Change: Human and Policy Dimensions*, *83*(102746), 102746.
